# Supplementary figures and images for: Meiotic prophase length modulates Tel1-dependent DNA double-strand break interference
Source: PLoS Genet. 2024 Mar 1;20(3):e1011140. doi: 10.1371/journal.pgen.1011140 (PMC10936813; doi:10.1371/journal.pgen.1011140)

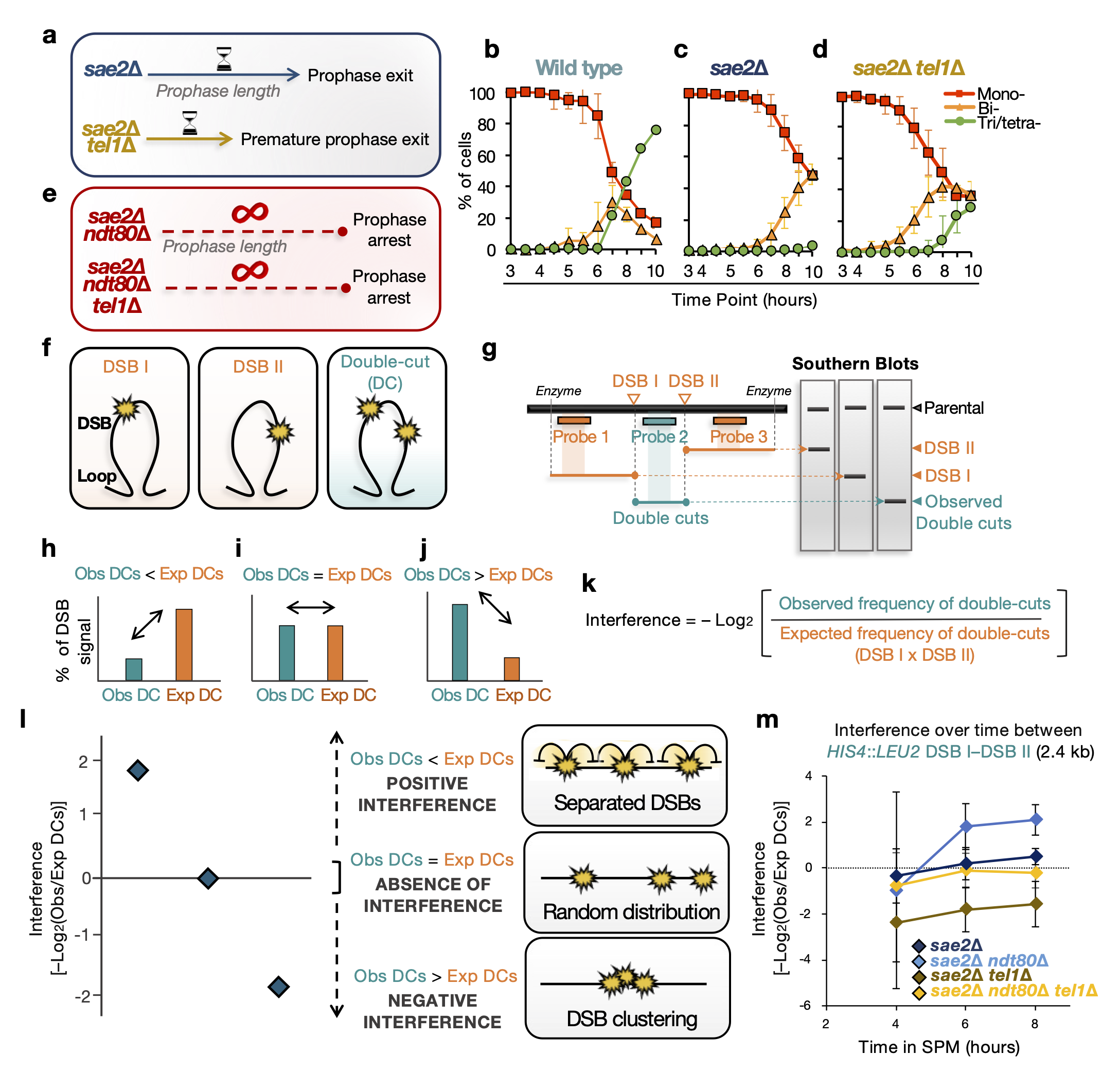

Supplement: S1 Fig — a, Schematic representation of the potential prophase length differences between ± Tel1. In the absence of Tel1, the checkpoint may be down-regulated resulting in a reduction of the meiotic prophase length. b–d, Meiotic nuclear division (MI and MII) kinetics showing the individual profiles of mono- bi-, tri/tetra-nucleate DAPI-stained cells for Wild type (b), sae2Δ (c) and sae2Δ tel1Δ (d). Summary of bi- tri- and tetra- previously presented in Fig 1B. e, Schematic representation of the expected effect of ndt80Δ mutation. Removal of NDT80 generates cell cycle arrest in late meiotic prophase I and therefore equalizes the length of meiotic prophase regardless of the presence or absence of Tel1. f–l, Simplified schematics of the Southern blot method used to study DSB interference at specific loci. f, Diagram representing a theoretical loop domain containing two hotspots (DSB I and DSB II) that can arise independently or coincidently (double-cut, DC). g, Diagram representing the position of the probes and fragments that would be used to detect each of the single DSBs or the coincident double-cut by Southern blotting techniques in this theoretical scenario. The probability of both DSBs arising from independence (Expected double-cuts), can be estimated by measuring and multiplying the single DSB event frequencies. h–j, Three possible scenarios can result from comparing the estimated expected DC frequency with the observed DC frequency. The expected DC frequency can be higher (h), similar (g) or lower (j) than the observed DC frequency. k, The strength of interference is calculated as the negative logarithm of the observed DC frequency divided by the expected DC frequency (obtained from the product of the two individual measured DSB frequencies). l, Positive interference values indicate separated DSB events. Interference values close to zero suggest absence of interference, and thus, potentially, a random distribution of DSBs. Negative interference values indicate concerted [file pgen.1011140.s001.tiff]

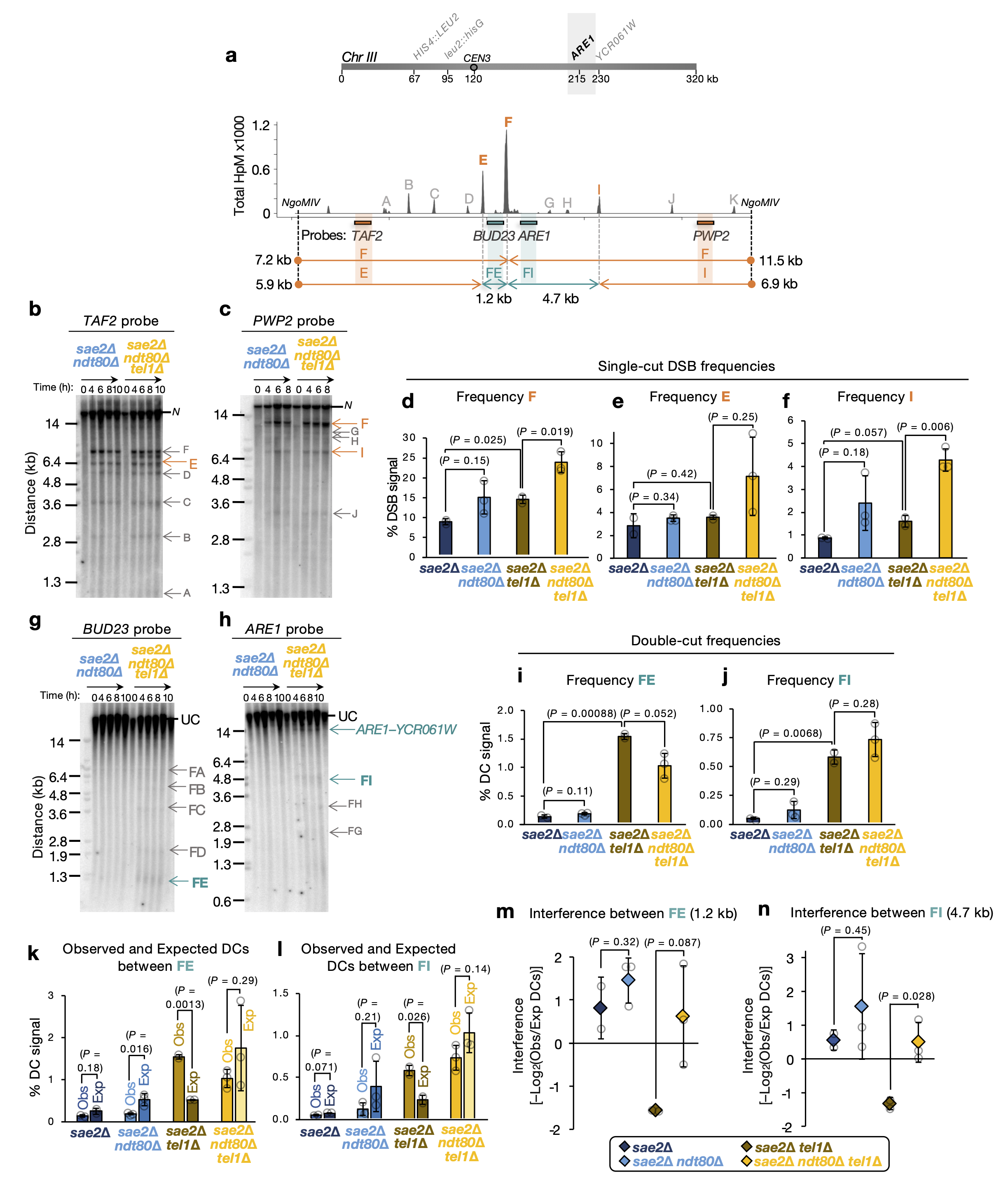

Supplement: S2 Fig — A, Top, Location of ARE1 region on chromosome III. Bottom, Diagram of the ARE1 hotspot showing Spo11-DSB positions as detected by CC-seq in hits per million (HpM; [38]), and, for Southern blotting experiments, the restriction enzyme sites, probes and size of fragments obtained from each probe. DSB interference was only measured between the main hotspot F–E and F–I. b–c, Representative Southern blots of genomic DNA isolated at the specified times hybridised with TAF2 (b), and PWP2 (c) probes. Quantified DSBs were marked in orange and not-quantified DSBs in grey. N, NgoMIV digested parental fragment. d–f, Quantification of F (d), E (e) and I (f) hotspots (average of 6–8 h time points). Estimation of F was corrected by adding on FI double-cuts measured with ARE1 probe. g–h, As in b–c but with undigested gDNA samples at the indicated timepoints and hybridized with BUD23 (g) and ARE1 (h) probes. Quantified DCs were marked in blue and not-quantified DCs in grey. UC, Uncut parental. i–j, Quantification of DC signal between FE (i) and FI (j) (average of 6–8 h time points). k–l, Quantification of observed and expected DC frequencies between FE (k) and FI (l) using averaged data from 6–8 h time points in the indicated strains. m–n, DSB interference between FE (m) and FI (n) calculated for each individual repeat expressed as–log2(Observed/Expected DCs) and then averaged (see Extended methods, “Calculation of DSB interference”). In all plots, error bars indicate Standard Deviation between individual repeats (overlaid grey circles on bar graphs). For statistical analysis, a two-tailed t-test with equal variance was performed with P values indicated. n = 2 for NDT80+ (from Garcia et al 2015 [56]) and n = 3 for ndt80Δ backgrounds. (TIFF) [file pgen.1011140.s002.tiff]

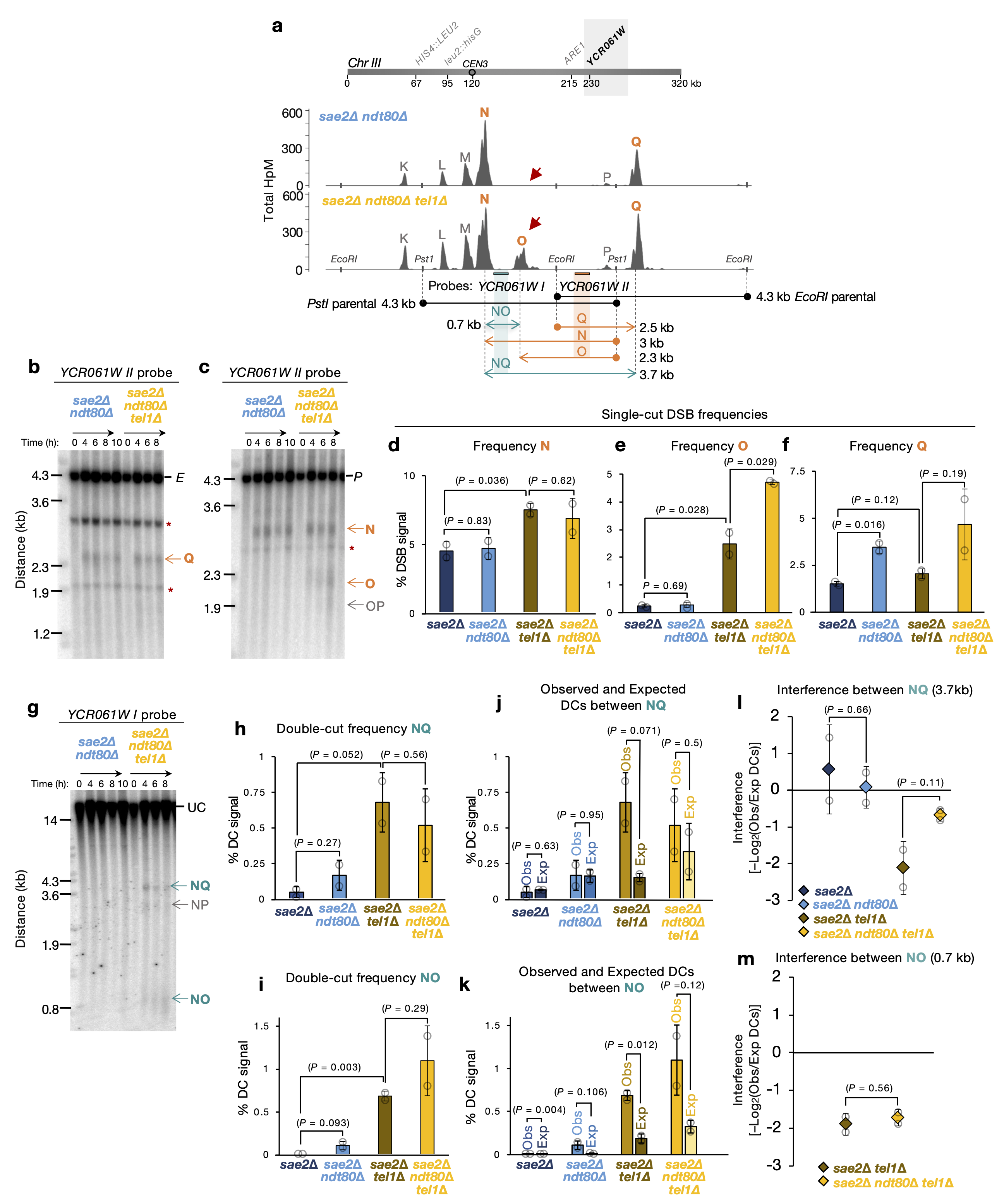

Supplement: S3 Fig — a, Top, Location of YCR061W region on chromosome III. Bottom, Diagram of the YCR061W hotspot showing Spo11-DSB positions as detected by CC-seq [38] in hits per million (HpM) and, for Southern blotting experiments, the restriction enzyme sites, probes and size of fragments obtained from each probe. DSB interference was only measured between the main hotspots N–O and N–Q. b–c, Representative Southern blots of genomic DNA isolated at the specified times hybridised with YCR061W II probe. E, EcoRI digested parental fragment (b) and P, PstI digested parental fragment (c). Quantified DSBs were marked in orange and not-quantified DSBs in grey. d–f, Quantification of N (d), O (e) and Q (f) hotspots (average of 6–8 h time points). Estimation of N was corrected by adding on NO DCs measured with the YCR061W I probe. g, As in b–c but with undigested gDNA samples at the indicated timepoints and hybridized with the YCR061W I probe. Quantified DCs were marked in blue and not-quantified DCs in grey. UC, Uncut parental. h–i, Quantification of DC signal between NQ (h) and NO (i) (average of 6–8 h time points). j–k, Quantification of observed and expected DC frequencies between NQ (j) and NO (k) using averaged data from 6–8 h time points in the indicated strains. l–m, DSB interference between NQ (l) and NO (m) calculated for each individual repeat expressed as–log2(Observed/Expected DCs) and then averaged (see Extended methods, “Calculation of DSB interference”). In all plots, error bars indicate Standard Deviation between individual repeats (overlaid grey circles on bar graphs). For statistical analysis, a two-tailed t-test with equal variance was performed with P values indicated. n = 2 for NDT80+ (from Garcia et al 2015 [56]) and for ndt80Δ backgrounds. (TIFF) [file pgen.1011140.s003.tiff]

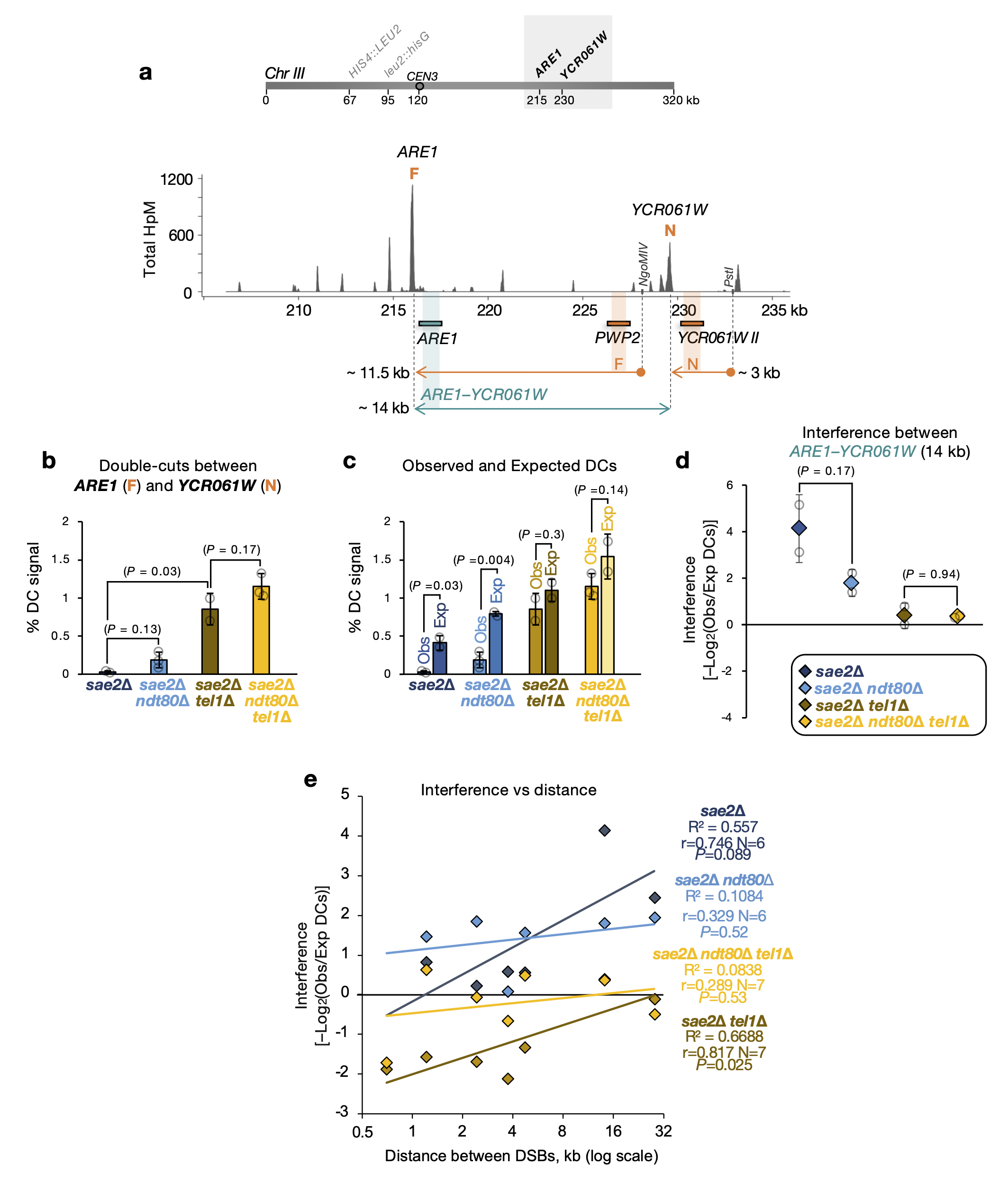

Supplement: S4 Fig — a, Top, Location of ARE1–YCR061W region on chromosome III. Bottom, Diagram of the region comprised between ARE1 and YCR061W hotspots showing Spo11-DSB positions as detected by CC-seq in hits per million (HpM; [38]), and, for Southern blotting experiments, the probes and size of fragments obtained from each probe. b, Quantification of F and N was obtained from S2C and S3C Figs, respectively. Quantification of DCs between ARE1–YCR061W was obtained from S2H Fig. c, Quantification of observed and expected DC frequencies between ARE1–YCR061W using averaged data from 6–8 h time points in the indicated strains. d, DSB interference between ARE1–YCR061W hotspots calculated for each individual repeat expressed as–log2(Observed/Expected DCs) and then averaged (see Extended methods, “Calculation of DSB interference”). In all plots, error bars indicate Standard Deviation between individual repeats (overlaid grey circles on bar graphs). For statistical analysis, a two-tailed t-test with equal variance samples was performed. n = 2 for NDT80+ (from Garcia et al 2015 [56]) and for ndt80Δ backgrounds. e, Aggregation of interference data from all 7 loci measured in this study. The mean value of interference was plotted against the distance (in kb) between the pair of DSBs used to measure interference on a log2 scale. R2, Pearson r, and P value of the Pearson correlation are indicated, highlighting the positive trends observed in NDT80+ strains that are substantially flattened upon NDT80 deletion. (TIFF) [file pgen.1011140.s004.tiff]

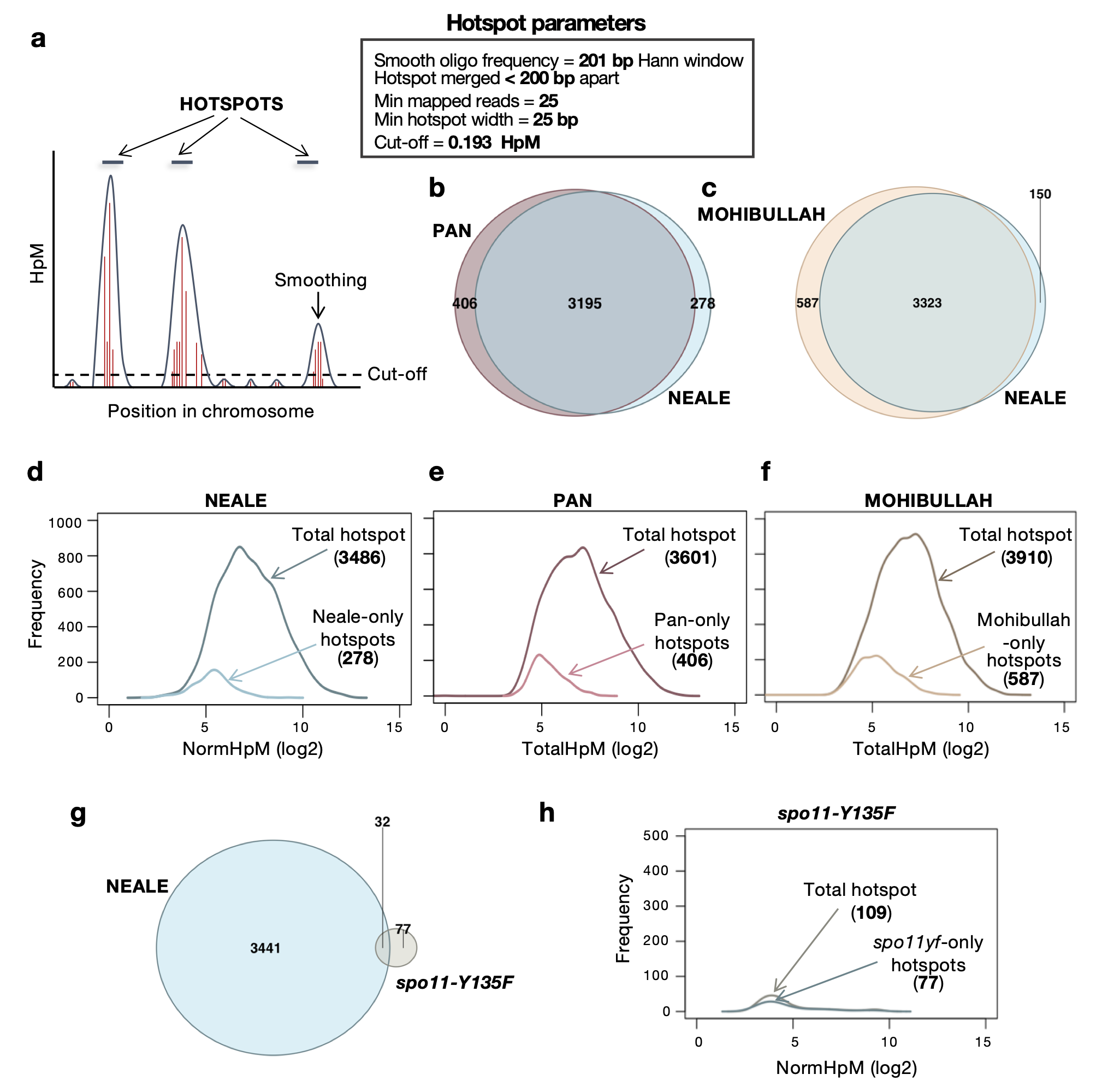

Supplement: S5 Fig — a, Diagram representing the hotspot calling method (see Extended method, “Hotspot identification”). The frequency of HpM was smoothed using a 201 bp Hann window with a minimum length of 25 bp, 25 reads and a cut-off of 0.193 HpM to filter for noise signal. Hotspots separated by < 200 bp were merged and considered as a single hotspot. In this study, hotspots were identified from a pooled combination of sae2Δ ndt80Δ and sae2Δ ndt80Δ tel1Δ (Neale template). b–c, Venn diagrams of overlap between hotspots identified in this study by CC-seq (Neale) and hotspots identified by Spo11oligo mapping by Pan et al. 2011 [40] (b) or Mohibullah et al 2017 [60] (c). d–f, Distribution of hotspot frequency strengths for the total and unique hotspots identified by Neale vs Pan (d), Pan vs Neale (e) and Mohibullah vs Neale (f). g, Venn diagrams of overlap between hotspots identified in the Neale template and the non-specific hotspots identified in the spo11-Y135F strain. The cut-off for hotspot calling in the sae2Δ ndt80Δ spo11-Y135F mutant was lowered to 0.125 HpM. h, as in d–f but sae2Δ ndt80Δ spo11-Y135F vs Neale template. (TIFF) [file pgen.1011140.s005.tiff]

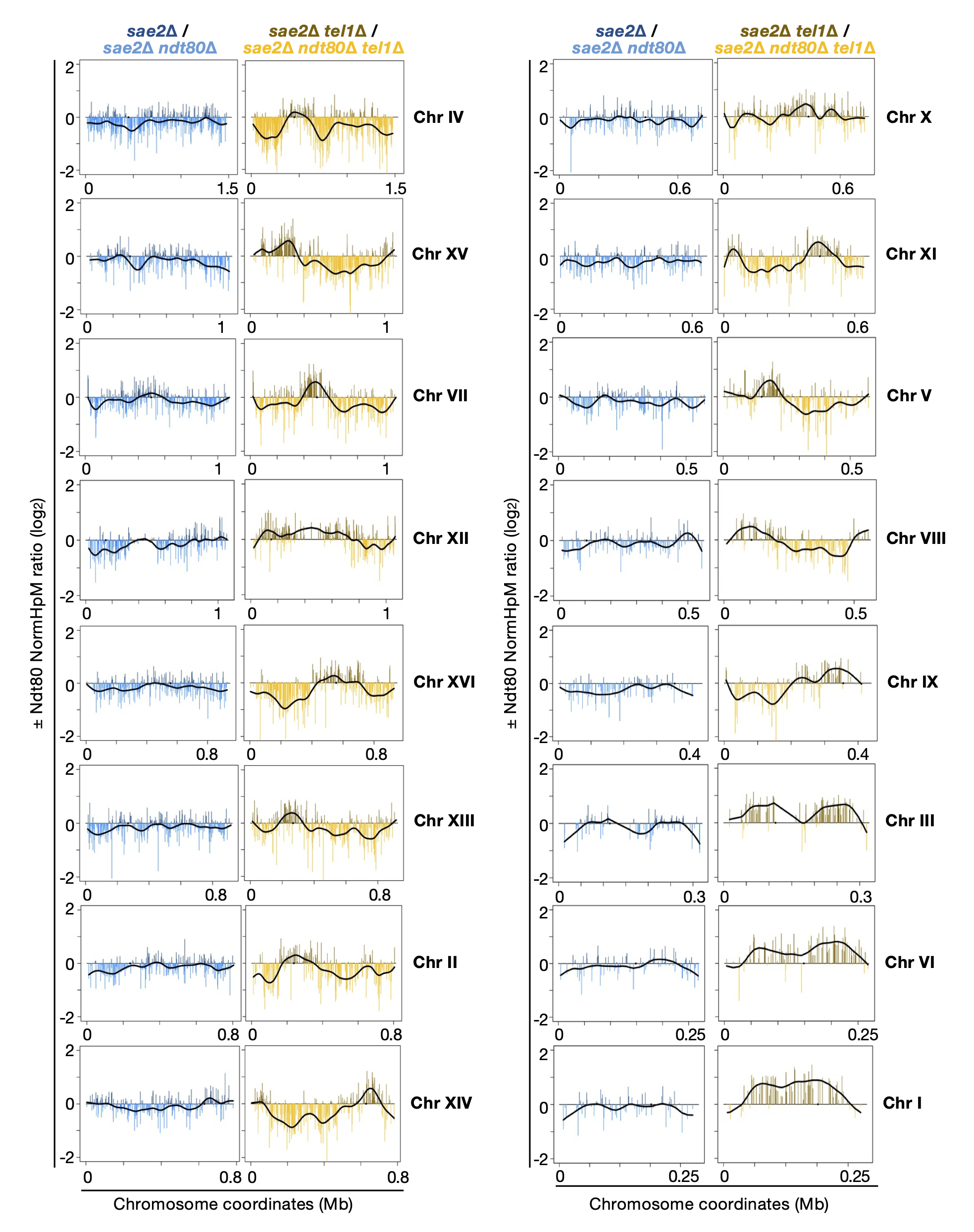

Supplement: S6 Fig — Log2 ratio of relative Spo11 hotspot intensities ±NDT80 on all 16 chromosomes in the presence (left panel) and absence (right panel) of Tel1. Values above zero indicate a higher DSB frequency in the presence of Ndt80 and below zero a higher DSB frequency in the absence of Ndt80. Fold change was smoothed to highlight the spatial trend effect of NDT80 deletion (black line). (TIFF) [file pgen.1011140.s006.tiff]

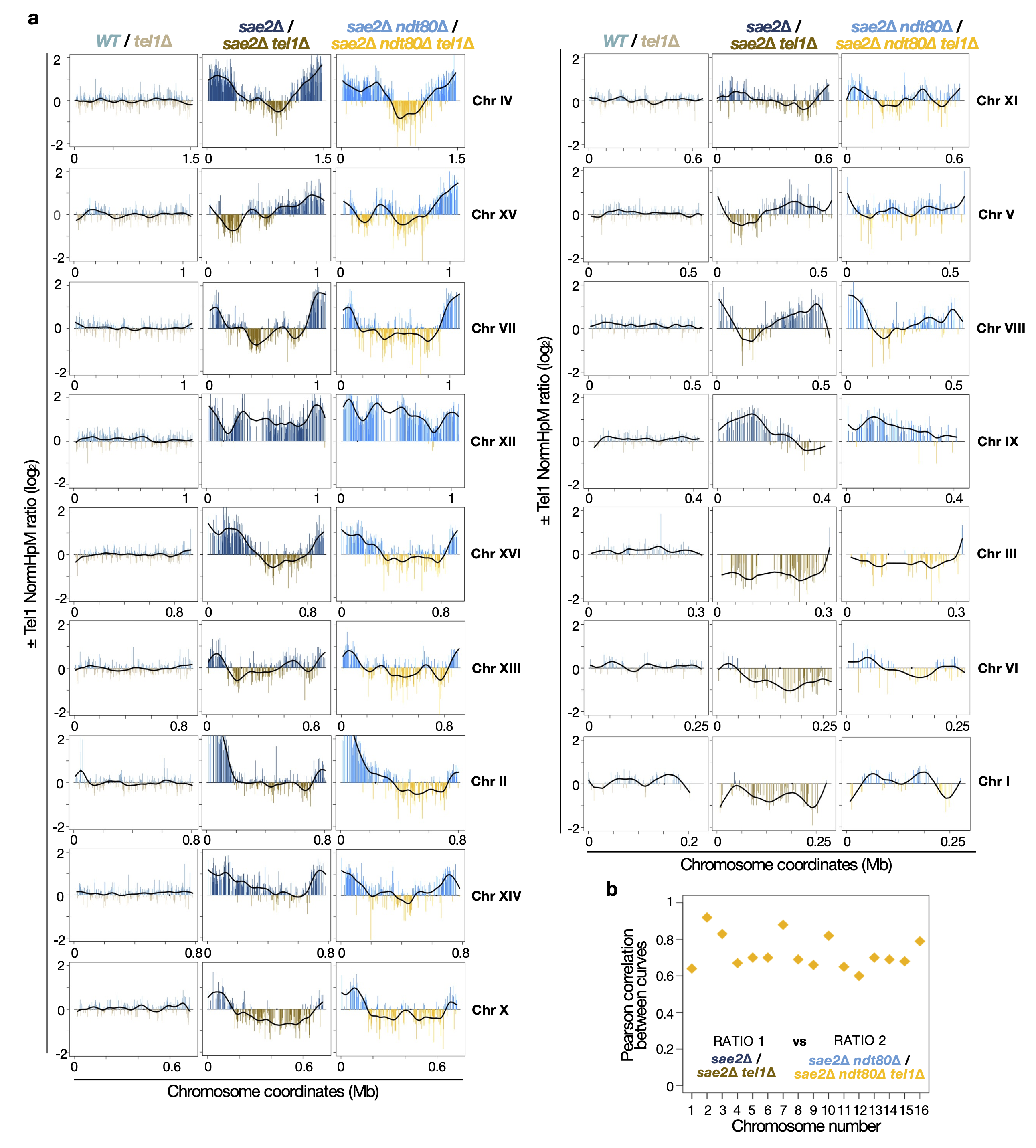

Supplement: S7 Fig — a, Log2 ratio of relative Spo11 hotspot intensities ±TEL1 on all 16 chromosomes in SAE2+ cells with Spo11-oligo technique (left panel) and sae2Δ cells with CC-seq technique in the presence (middle panel) and absence (right panel) of Ndt80. Values above zero indicate a higher DSB frequency in the presence of Tel1 and below zero a higher DSB frequency in the absence of Tel1. Fold change was smoothed to highlight the spatial trend caused by TEL1 deletion (black line). b, Plot showing the Pearson correlation between ± Tel1 smoothed ratios in the presence (RATIO 1) and absence (RATIO 2) of Ndt80 for each chromosome. (TIFF) [file pgen.1011140.s007.tiff]

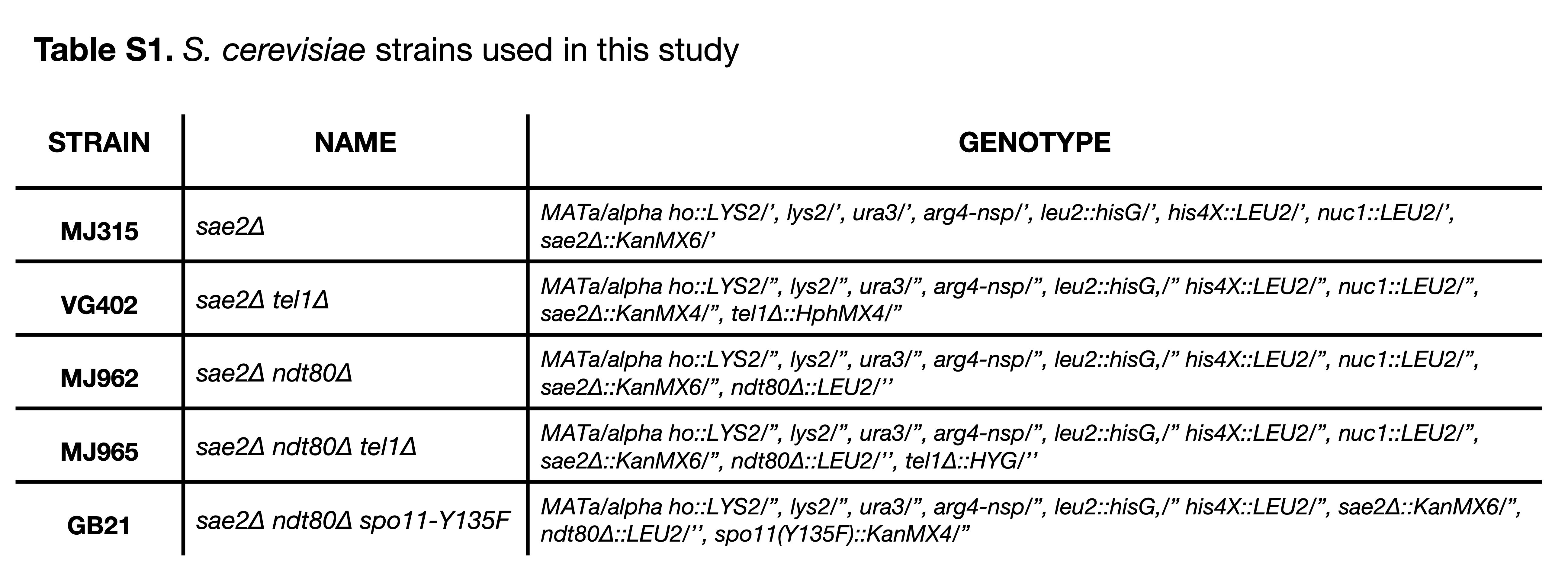

Supplement: S1 Table — All genotypes are otherwise isogenic from the SK1 strain background. (TIFF) [file pgen.1011140.s008.tiff]

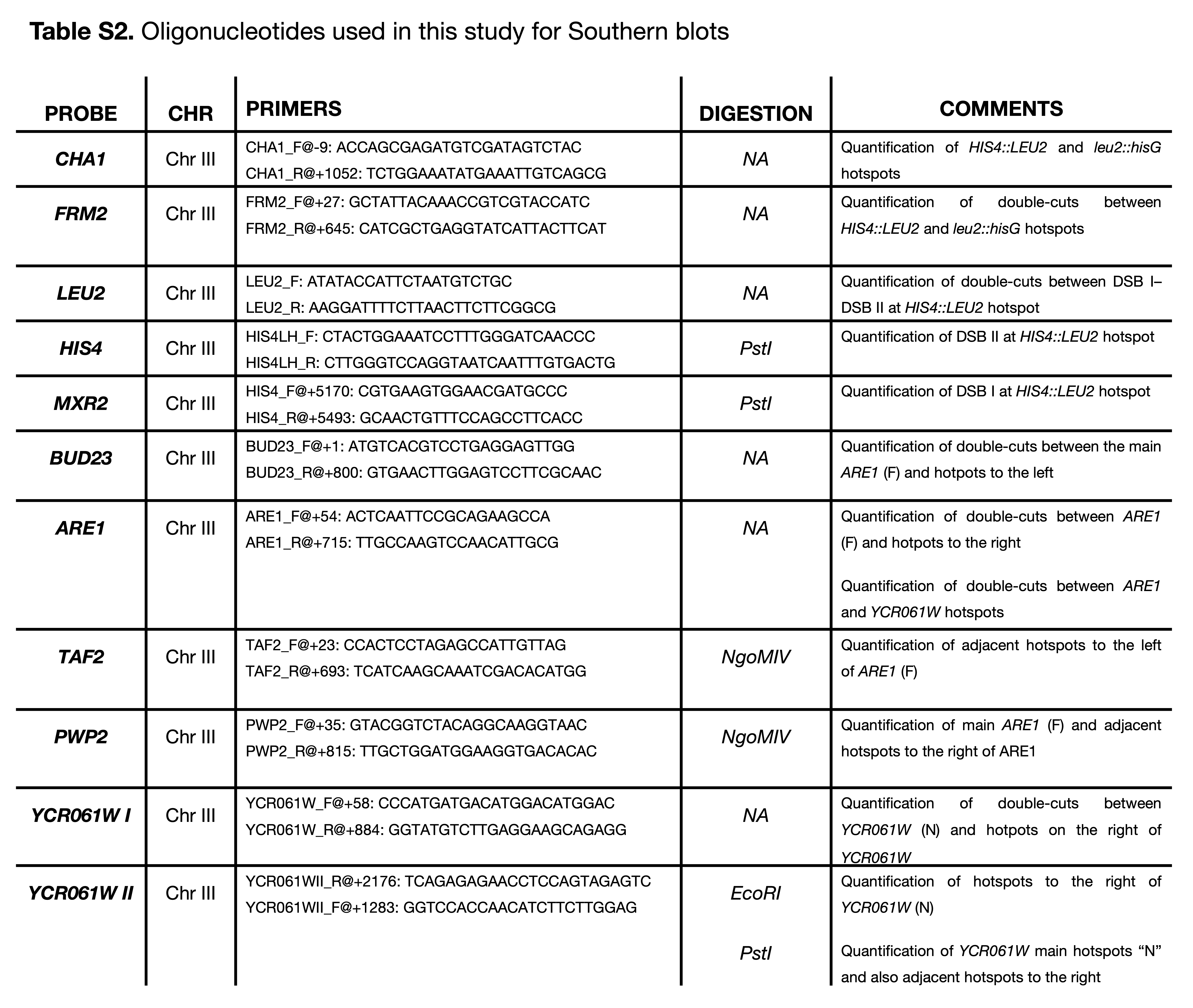

Supplement: S2 Table — Oligonucleotide pairs were used in PCR to generate locus-specific probes for Southern blots. CHR indicates chromosome. PRIMERS indicate locus name and DNA primer sequence. DIGESTION indicates whether the probe was used for digested or undigested DNA (with relevant enzyme as applicable). COMMENTS indicates relevant information for this probe and/or digest combination with respect to data collection within this study. (TIFF) [file pgen.1011140.s009.tiff]

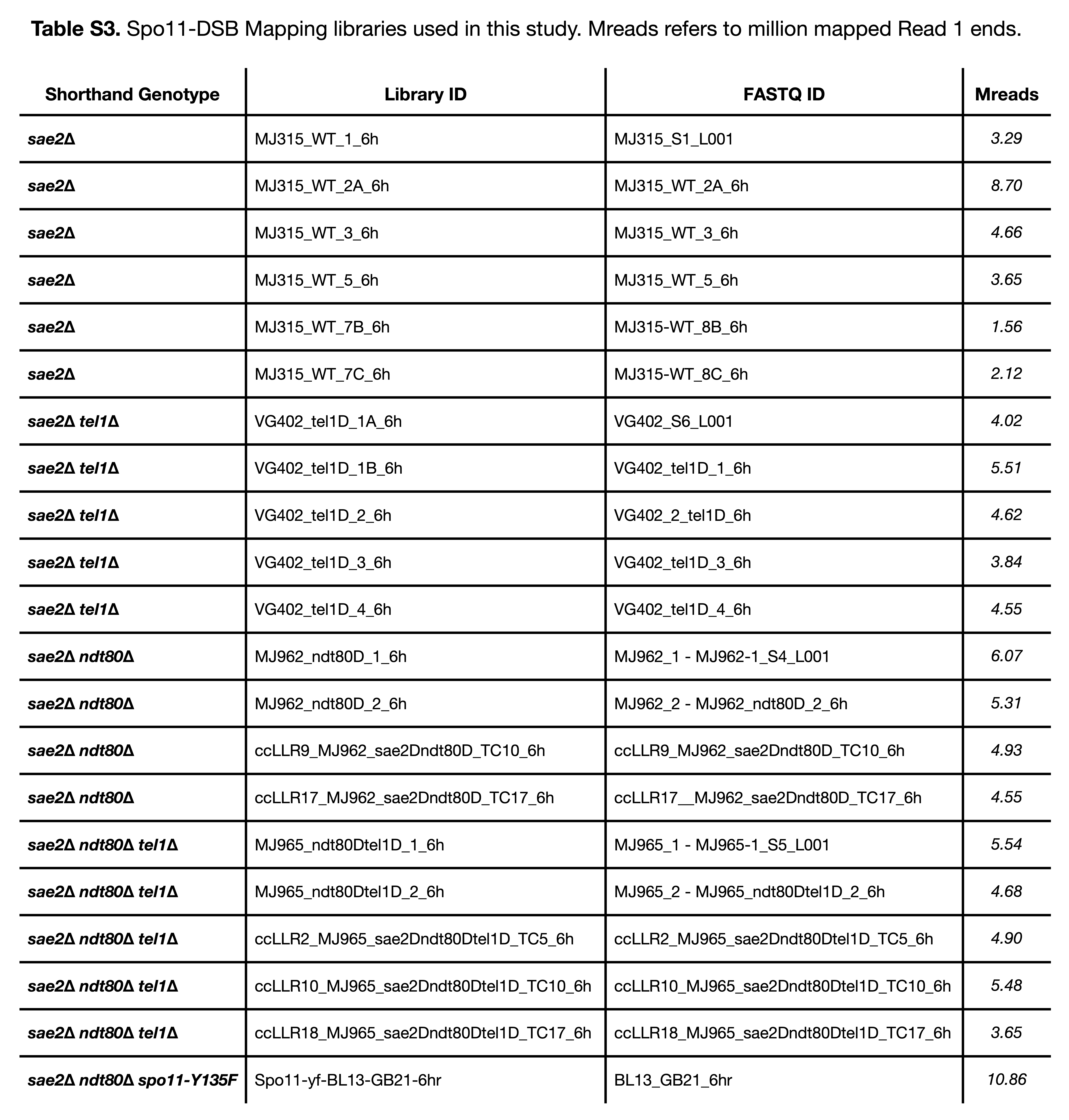

Supplement: S3 Table — Mreads refers to million mapped Read 1 ends (the Spo11-bound CC end). For pooled data, identical genotypes were averaged with equal weighting of each library. (TIFF) [file pgen.1011140.s010.tiff]

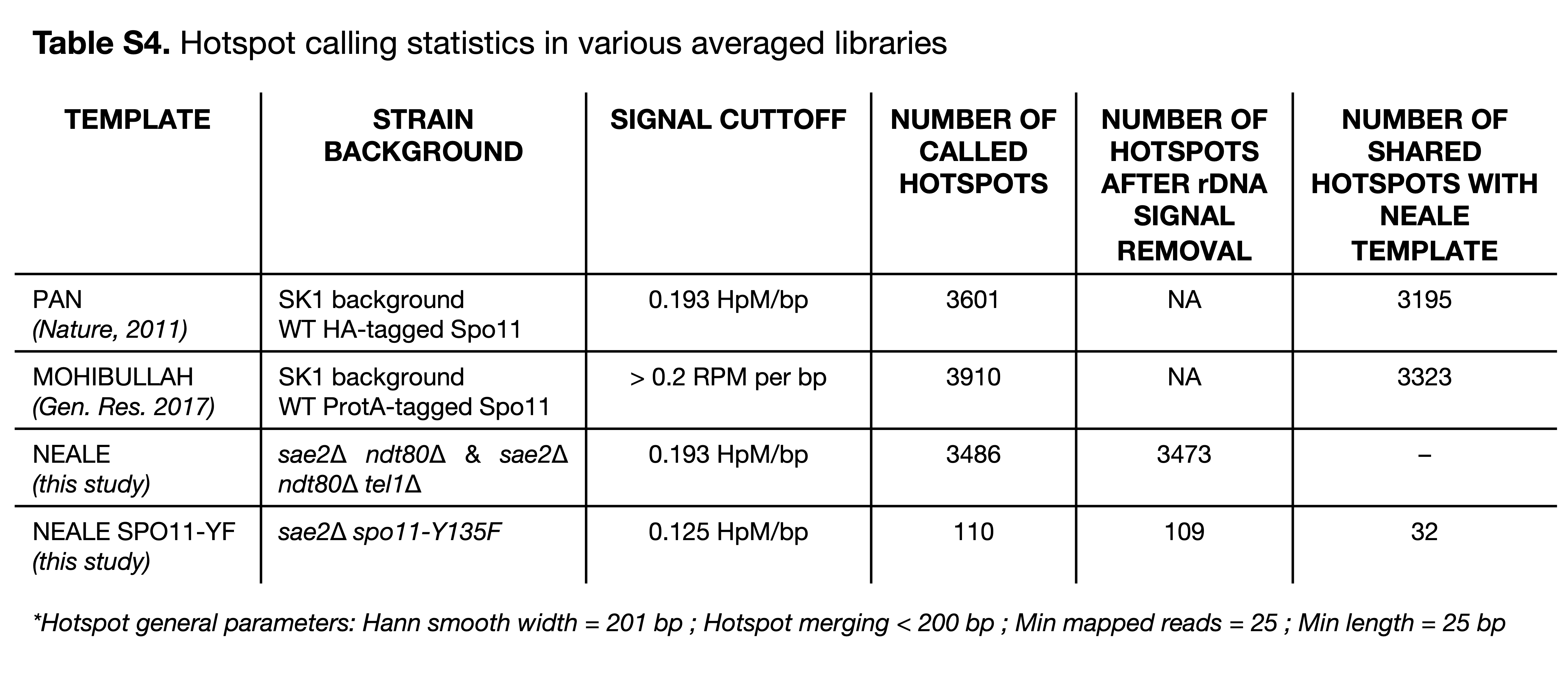

Supplement: S4 Table — (TIFF) [file pgen.1011140.s011.tiff]
