# Supplementary material for: Meiotic prophase length modulates Tel1-dependent DNA double-strand break interference
Source: PLoS Genet. 2024 Mar 1;20(3):e1011140. doi: 10.1371/journal.pgen.1011140 (PMC10936813; doi:10.1371/journal.pgen.1011140)
Supplement: Attachment — Submitted filename: PLoS Reviewers Responses to QuestionsB.pdf [file pgen.1011140.s013.pdf]

PLoS Reviewer's Responses to Questions

We thank the reviewers for their detailed and constructive comments. Our responses are interleaved in blue text below.

REVIEWER 1:

Reviewer #1: The manuscript by Ruiz et al. is a rigorous and elegant study of a type of DSB feedback control previously defined as "DSB interference", where a given DSB formation prevents other DSBs from forming nearby. In the previous study, they developed a method to measure the interference level using the ratio between the observed and expected frequency of chromatids cleaved twice (double cuts: DCs). Using this approach and the tel1Δ mutant, they demonstrated that DSB interference requires Tel1. They also discovered that the interference level varies depending on the inter-hotspot distances. Between hotspots ~20 kb apart, the TEL1 cells form fewer DCs than expected (positive interference), whereas the tel1Δ mutants form DCs equivalent to expected (no interference). In contrast, within short ranges (<15 kb), TEL1 shows no interference, while tel1Δ forms DCs more frequently than expected (negative interference, i.e., DSB clustering). To explain the negative interference in tel1Δ, they proposed that these neighboring DSBs reside within one of the DSB-permissive domains subject to stochastic activation during prophase. They demonstrated by a simulation that the decrease in the frequency of the DSB-permissive domain would underestimate the interference levels, leading to negative interference.

This study by Ruiz et al. experimentally tests the above scenario. The absence of Tel1 leads to premature prophase exit that terminates global DSB formation, potentially decreasing the frequency of activating the chromosomal sub-domains. If so, eliminating the premature prophase exit in tel1Δ would equalize the number of the DSB-permissive domains with that in TEL1, thereby attenuating the negative interference. To this end, the authors used the ndt80Δ mutant, which arrests cells in prophase. Consistent with the previous hypothesis, ndt80Δ eliminated the negative interference between DSBs at short distances in tel1Δ and led TEL1 cells to exhibit positive interference. In contrast, medium-range interferences are not affected by ndt80Δ. Furthermore, the authors used CC-seq, developed in a previous study, to visualize the effect of Tel1 and Ndt80 on the genome-wide distribution of DSBs and showed that chromosomal regions that preferentially form DSBs in tel1Δ correlate with the early association of proDSB factors, consistent with the expected depletion of late-forming DSBs in tel1Δ due to the premature prophase exit.

Overall, the paper is well written, the experiments are well designed, and the data broadly supports the conclusions that Tel1 mediated interference suppresses DCs within short and medium chromosomal range. Especially the experimental validation of the negative interference strengthens their fascinating proposal of stochastic activation of chromosomal sub-domains. Taken together with CC-seq maps elucidating the genome-wide impacts of Tel1 and Ndt80 on DSB distribution, this study further articulates how DSB interference and prophase length control mediated by Tel1 operate throughout meiotic prophase and shape the DSB landscape.

General comments:

I encountered difficulties in following how negative interference relates to the chromosomal sub-domains that form DSBs preferentially in the tel1Δ mutant. I thought that the premature exit from prophase would lead to a reduction in the frequency of activating sub-chromosomal domains, particularly in regions where DSBs occur later, thus further tilting the interference measurement towards negative values.

We think there are three aspects influencing the observations:

1. We infer that subdomains arise both in the presence and absence of Tel1 (both *sae2Δ* and *sae2Δ tel1Δ*), due to limited opportunity to activate all regions.
2. Additionally, in the absence of Tel1, cells progress faster through prophase, exacerbating this effect, limiting the opportunity to activate domains further.
3. Finally, due to Tel1's role in mediating DSB interference, the effects manifest as strong negative interference in the absence of Tel1.

In terms of early vs late regions, indeed it is possible that later regions are also (or even more) subject to the subdomain effect, however, the fact that we can already observe these effects in the early Rec114-associating regions suggests that the subdomain effect arises even in early regions. Deletion of *NDT80*, then appears to resolve these subdomain effects by giving more time/opportunity to generate active regions.

In addition, I noticed that the hotspots examined in the paper are exclusively located in chrIII, where Rec114 is associated earlier. It would be beneficial if the authors could clarify the above and explain their decision to present this specific subset of hotspots, especially considering that their previous paper includes hotspots on other chromosomes.

We focused our attention on these hotspots because they were easier to study (well defined hotspots) and they were used in the previous study to clearly characterise the effects of interference. We did indeed also attempt to analyse DSB formation, DC formation and DSB interference at hotspots located in different chromosomes [*SRB2* (in chromosome VIII) and *DOT5* (in chromosome IX)], however, despite repeated attempts, the Southern blot quality was not good enough to be analysed.

We did measure DSB formation (but not DSB interference) at some other strong hotspots (*CCT6*, *RSN1*, *ERG25* and *GAT1*) but only in the *sae2Δ ndt80Δ* background, so we could not compare these values with the *sae2Δ* data.

To recognise these points, we have added a paragraph in the discussion highlighting that the DSB interference measurements are restricted to chromosome III, and have discussed the relationship to early vs late Rec114 binding: (Line 566-577)
"Our study was also limited to measuring DSB interference only between the strong hotspots on chromosome III. Whilst attempts to characterise DSB interference at other genomic loci were made (as performed in prior work{Garcia et al., 2015, #16838}), the resulting data displayed too much technical variation to enable useful analysis. It is therefore important to recognise that the DSB interference measurements—and the changes we observe upon *TEL1* and *NDT80* deletion—are limited to regions of the genome that, based on Rec114 association timing{Murakami et al., 2020, #60734}, are likely to generate DSBs earlier than the genome average. However, whether it is the Rec114 timing difference itself—or some other feature of

DSB regulation—that underpins the inferred subpopulation timing effects will require further investigation. For example, it is reasonable to expect that genomic regions that load Rec114 later are also (or even more) subject to subdomain effects due the fact that relatively few cells in the population may have an opportunity to prime such genomic regions for DSB formation."

Specific comments:
L56: ref38 uses SAE2+ and is inappropriate in this context.
We have removed this reference.

L92: Homolog engagement down-regulates DSB formation in a chromosome-autonomous manner. "global" makes me feel awkward. I also suggest citing ref 90 as well.
"global" was removed and X. Mu et al 2020 was added. (Line 92-94)

L901-904: I wonder if the authors had explained that this correction is reasonable in the previous paper. If so, cite the paper here.
Yes, this correction was also described in the Methods of Garcia et al 2011, section: "**DSB analysis by Southern blotting:** Analysis of double-cutting at the *HIS4*::*LEU2* locus (Fig. 3) necessitated taking into account of the fact that the strains used in this study contain three copies of the *LEU2* gene: at the *his4X*::*LEU2*, *leu2*::hisG and *nuc1*::*LEU2* loci. Specifically, because the probe designed to detect double-cuts between sites I and II within the *his4X*::*LEU2* hotspot recognizes these three loci, numerous cross-reacting bands arise when prob- ing DNA digested with restriction enzymes. Therefore undigested DNA was fractionated and transferred as for other loci. However, double-cut values recorded by this method were multiplied by three to correct for the fact that only 1/3rd of the uncut parental DNA signal originated from the *HIS4*::*LEU2* locus. "

We have added a citation to this prior work in the Extended methods section (Line 799).

Figure 1l: I wonder if the authors could provide the numbers from replicates that were used to generate this figure panel as supplement data to help readers understand how these numbers are transformed into interference in Figure 1m.

We have provided a Supplementary excel file which includes the individual repeats (average 6-8h) for figure 1l and m. "HIS4LEU2_FIg1lm_ForReview.xlsx"

Figure 1m: It may be worth presenting the kinetics of interference over 4-6 hours. The trend that is similar to Extended Data Figure 3i in the previous paper (Garcia et al., nature 2015) would further support the authors' model.

We have added an additional panel to Fig S1 (Fig S1m) detailing the change in interference values over time (within error, it doesn't substantially change), and have referred to this in the main text. (Line 198-200 and 217-219).

Figure 3a: I am curious if it is possible to calibrate CC-seq datasets to represent absolute DSB levels, for example, by quantifying the amount of the "Sonicated Spo11–DNA" or using DSB frequencies detected by Southern blotting.

In principle it is possible to estimate absolute differences in DSB frequencies in different strains/libraries by adding an exogenous DNA spike-in to compare with. For this we now use DNA obtained from human cells treated with etoposide (i.e. containing lots of Top2-CCs). However, most of the datasets used within this manuscript were prepared prior to developing the spike in, and thus do not contain it.

We separately have also explored calibrating the CC-seq maps by normalising to %DSB breakage at several strong hotspots (*HIS4*::*LEU2*, *YCR061W*, *RSN1*, *GAT1*, *ERG25* and *CCT6*) as detected by Southern blotting (similar to as was performed by Pan et al, 2011), however, the experiments have only been performed in the *ndt80*Δ background, and, so far have proved rather variable, so we do not feel comfortable using these as a way to calibrate the data, and thus feel more confident in focusing on changes in spatial patterns along and between chromosomes that by their nature are inherently internally controlled.

Figure 3g-h: The visual correlation (e.g., stated in L449) lacks quantitative perspectives. I suggest adding scatter plots with correlation coefficients comparing panels 3g and 3h as well as others (e.g., 4f and 3h seem to correlate).

We have performed a statistical analysis to plot the spearman correlation between each bin for each comparison(3i & 4i) and described these in the text (Line 342-351): Panel 3f vs 3h and 3g vs 3h. Also 4f vs 4h and 4g vs 4h (Line 397-405). We have also replotted Panel 3h in panel 4h to enable better visual compassion within Figure 4 and have stated this in the text..

Figure 3h: The authors should explain how they converted the original data to generate this figure panel somewhere.

The Rec114 accumulation timing data was downloaded from the original paper (GSE52970_tRec114_A+). These data were filtered to remove rows marked in the table (described in the manuscript as having low confidence), smoothed using a loess curve (normalised for chromosome length), and segmented into 50 kb non-overlapping windows. The units are in hours and we have now represented the values on a red–white–blue colour scale from 2.3 (early) to 2.8 (late) hours.

We have added this information to the legend and methods section (Line 785-789).

Figure 5: I thought the interference measurement skews towards negative because tel1Δ activates fewer domains, such as illustrated in their previous paper (Extended Data Figure 8 in Garcia et al., nature 2015). However, in Figure 5a of the current paper, TEL1+ and tel1Δ

show the number of active domains. The authors might intend to convey different messages, but the current figure confused me. In addition, I found the numbers simulation presented in the previous figure helpful in following the logic. Therefore, I wonder if the authors could provide a similar simulation.

We have revised Fig 5 to make it comparable to the presentation in Garcia et al. 2015, but with a parallel simulation (Fig 5b) for the effect on interference calculations in the absence of Ndt80, which we infer removes the subpopulation effect, and thus no longer causes negative interference to be calculated/observed.

Supplementary Figure 1k: This definition of interference leads to different numerical behavior when given DSB pairs exhibit positive and negative interference (panels h and j), making it difficult to imagine the ratio between observed and expected DCs. If the authors are not strongly committed, they could change the definition of interference to straightforward ones (e.g., -log2(obs/exp)).

We thank the reviewer for this excellent suggestion. We now represent interference via the equation: -Log2(Obs/Exp) and explain this in the methods (Line 826-834), thereby avoiding the prior compression of positive interference values between 0 and 1. In addition, as suggested by Reviewer #2, we have also now plotted individual data points on every graph to enable readers to appraise the degree of variation within our dataset.

Supplementary Figure 1k legend: Please, fix typos.
We have corrected this error.

REVIEWER 2:

Reviewer #2: During meiosis the introduction of DNA double-strand breaks (DSB) by Spo11 initiates homologous recombination. This process needs to be tightly regulated to ensure that DSBs form in a spatially and temporally coordinated manner throughout the genome. One key regulator of meiotic DSB formation is Tel1/ATM kinase. Two distinct roles for Tel1 that are important for the understanding of this manuscript have been characterized previously by the authors and other groups. First, Tel1 mediates DSB interference locally (across distances of 70-100 kb) through the inhibition of breaks in adjacent DSB hotspots. DSB interference influences DSB patterns and also limits overall DSB numbers. Second, Tel1 regulates DNA damage checkpoint activation and delays exit from prophase I, which is initiated by expression of the transcription factor Ndt80. How these two roles affect each other is unclear.

To address this question the authors characterize DSB formation using a series of S. cerevisiae mutant allele combinations: sae2Δ, tel1Δ, and ndt80Δ. While the phenotype of tel1Δ in the sae2Δ background has been studied before, additional deletion of ndt80Δ now allows the authors to study the effects of Tel1 in more homogeneous populations of cells that arrest in late Prophase, thereby removing the potentially obscuring effect of premature exit from

Prophase due to the lack of checkpoint activation in tel1 mutants. The authors use Southern blot assays together with genomic approaches to characterize DSB formation at different scales: overall DSB formation within a series of different hotspots, DSB interference between closely adjacent hotspots (ca. 0.7-3 kb) or across medium distances (ca. 15-30 kb) and lastly genome-wide DSB patterns.

They find that an extended Prophase due to ndt80∆ leads to a variable increase in DSB formation in the absence of Tel1, suggesting that the total DSB potential in the absence of Tel1 might have been previously underestimated, at least for some genomic loci. Interestingly, the effect of ndt80∆ on interference differs substantially between very close hotspots and across medium distances. The effect of Tel1 loss at very close hotspots (loss of interference and clustering of DSBs) is dependent on Ndt80, while at medium distance ndt80∆ has no effect. Global DSB landscapes show some remodeling in the absence of Ndt80, an effect that is stronger in the absence of Tel1. In ndt80∆ strains the increased DSBs form in regions normally less prone for DSB formation, making the overall DSB hotspot strength more homogenous across chromosomes. This is consistent with a model were shorter Prophase length (in the presence of Nd80) in tel1∆ leads to preferential DSB formation in very hot regions that previously were correlated with early and long Rec114 association time. Lastly ndt80∆ allows the authors to characterize the effect of tel1∆ genome-wide in a population of cells without premature exit from Prophase, showing that in a sae2∆ background the presence of Tel1 changes global distribution of hotspot strength across chromosomes in a largely Ndt80-independent manner.

This study addresses important, longstanding questions in the field and it provides several interesting observations and broadly useful datasets. The data are clearly presented and there are well-reasoned implications discussed for understanding how DSB patterning within individual cells plays out when measured across large populations of cells.

We thank the reviewer for these positive comments on our manuscript.

However, there are also several significant limitations to the work in its present form that need to be addressed. The main concerns are a) there are insufficient numbers of replicates for most of the Southern blotting assays, resulting in experiments that are underpowered to detect differences rigorously; b) the analysis of the whole-genome data is somewhat superficial and limited in scope; and c) an important caveat with the use of the sae2 mutant background needs to be acknowledged.

a) We thank the reviewer for their detailed assessment. Regrettably, there is indeed often quite a lot of variance in the DSB measurements which we think are largely due to the technical limitations of the Southern blotting method. We had presented the error of the mean and P-values of a two-tailed test for the Southern blots to allow the reader a way to appraise the differences we observe. To further aid this point, in our revised submission we have overlaid individual DSB and DC measurements as hollow circles in addition to the average bar, and changed to standard deviation (SD). We have also removed “ns”, which we think is unnecessary and potentially misleading for P-values that were sometimes only modestly above 0.05. (In many instances, the P-values become “significant” if we had chosen a one-tailed test (to test only for increases for example). We actually think that the strength of our

data is that we are able to draw conclusions in aggregate despite the relatively high degree of variance of some individual measurements.

We would like to emphasise that the critical part is that over short distances there is a general trend towards strengthening of interference when *NDT80* is deleted (towards positive in *TEL1+* and from negative to no interference in *tel1*Δ backgrounds). To aid this point, we have added statistical tests to the observed/expected graph further supporting our interpretation that interference is positive in *sae2*Δ *ndt80*Δ (because Exp>Obs), and negative in *sae2*Δ *tel1*Δ (because Obs>Exp), but that observed and expected values are similar in the *sae2*Δ and *sae2*Δ *tel1*Δ *ndt80*Δ strains.

Ultimately, we are presenting what we feel is a plausible explanation and model based on assimilating all the observations we have made in aggregate (including the genome-wide changes in DSB distribution ±Ndt80 ±Tel1). In combination, we think the data are coherent, and strong enough to support our model and interpretation.

Specifics about data collection via Southern blotting: For the primary data—at *HIS4*::*LEU2*—we have presented a high number of replicates. For the secondary hotspots (presented in supplementary figures), there are always at least two replicates. Whilst we acknowledge that this may mean that addressing quantitative differences is sometimes underpowered, on balance we feel that the data presented are broadly supportive of one another, showing similar trends—which we feel is the important point, rather than being able to obtain precise values at every single locus, which we feel is beyond the scope of what is already a detailed study incorporated a very large amount of data collection over many years. From a technical viewpoint, many more experiments and gels were attempted, however, due to technical complexities of Southern blotting, they are often unsuitable for quantification, limiting us to use those observations where we feel relatively accurate measurements can be made. Broadly speaking, when measuring DSB frequencies, and especially the weak double-cut frequencies, signals are often close to the limit of measurability, and I would be concerned to read too much into precise values at any one location (in any study) even if it were to be repeated many many times.

We have acknowledged these limitations in a section of the methods titled: “ Limitations of the Southern blot and pulsed-field gel electrophoresis techniques”

We have addressed the other points as they are raised below.

Specific comments

1. There are a number of places where differences between genotypes are highlighted despite those differences not being statistically significant. Specifically:

- Line 246: Since the difference between sae2 tel1 and the triple mutant is not statistically significant (Fig. 2c), it is not appropriate to state that the tel1 and ndt80 effects are additive.

Lines 252-253: the ndt80 deletion did not have a statistically significant effect in either the TEL1+ or tel1 background.

We have reworded this section to tone down our description (Line 256-262), and explicitly state the P values reported in the (updated) figure to help the reader draw their own conclusion. We have also added a closing sentence to help emphasise and summarise the findings of this figure with respect to interference measurements over medium range and the apparent lack of an effect of *NDT80* deletion.

- Line 261: the text says that DC frequencies are increased in the ndt80 mutant in both the presence and absence of Tel1, but the results of a statistical test are not provided in Fig. S4b (unlike in other figures).

We have added P values to Fig S4b and amended the text similar to above (Line 264-268) to acknowledge that the increases have marginal statistical significance (at least when using a two-tailed test). We note that had we elected to use a one-tailed test (to test for increases only), the increases we observe would be very close to a significance threshold of P=0.05.

- Line 372-374: This statement ("both TEL1 and NDT80 deletion independently increased Spo11 activity, with the greatest DSB frequency arising when both genes were deleted") is only true for some of the measurements, but is not universally correct.

We have revised this sentence (Line 427-430) to read: "When considering total DSB levels, whereas TEL1 deletion alone increases DSB formation, the effect of deleting *NDT80*—and thus of extending prophase—was generally stronger only in the absence of Tel1 (Fig 1f-h; Fig S2d-f; Fig S3d-f), suggesting that Tel1 activity becomes even more critical in limiting DSBs in an ndt80Δ background."

2. The above issues need to be addressed by appropriate rewriting to avoid claims based on differences that are not statistically significant. However, a bigger issue is that the measurements are intrinsically somewhat noisy, but the number of replicates was low (often just 2), so the study is underpowered to detect differences if they are there. It is essential to have more replicates to address these questions rigorously.

To address this point we have amended the text in the places pointed out above as suggested, we have also included P values in the text and figures (where they were missing), we now present error bars as standard deviation, and we present each individual data point as a circle overlaid on the bar to enable readers to directly appraise the degree of variance in individual measurements. Whilst we acknowledge that some of the measurements, by the nature of the Southern blotting assay, are somewhat noisy at any individual locus, we feel that when taken as a whole, the dataset is coherent and enables us to present a rational message regarding the effects of *NDT80* deletion on measurements of Tel1-dependent interference.

3. The CC-seq datasets are an important addition to the field, and several interesting observations are made with them. However, the overall analysis is some limited in scope, with only fairly limited analysis of chromosomal or chromatin features that might correlate with the

observed changes. A more comprehensive analysis is warranted to determine what might be driving the variation across the genome for the effects of the tel1 and ndt80 mutations.

We thank the reviewer for their positive comments. As also suggested by Reviewer 2 we have mentioned the association with centromeric and telomeric regions and have added quantitative correlations between the fold-changes caused by either *NDT80* or *TEL1* deletion and the timing of Rec114 association. We have also included a section in the discussion concerning the relative paucity of signal on chromosome XII in *sae2*D *tel1*D cells (Lines 539-545). Furthermore, a manuscript specifically focusing on the Tel1-dependent changes (itself a major study) is currently in preparation. We feel that in order to stay focused on the main message of the paper (the *ndt80* effects on interference) that the depth of analysis that we have performed is appropriate. Of course, all CC-seq datasets will also be made available on publication for other researchers to probe. (Line 342-351): Panel 3f vs 3h and 3g vs 3h. Also 4f vs 4h and 4g vs 4h (Line 397-405)

4. Although the use of the sae2 mutant for these studies is reasonable, using this background introduces caveats that make it challenging to extrapolate to the situation in a SAE2 background. The authors acknowledge one such caveat (line 424; and lines 471-477), having to do with the suppression of the normal DSB response in sae2 meiosis because of absence of the ssDNA that is normally generated by resection. Additionally, however, it has been established that sae2 mutants, in addition to failing to initiate DSB resection, are also constitutively hyperactivated for Tel1 activity, at least in vegetative cells (PMID: 11430828, 18245357). If this hyperactivation is also true in meiosis, then the sae2 background would be expected to artificially exacerbate the effects of a tel1 deletion compared to a SAE2 background. This issue needs to be discussed, including acknowledgment of the possibility that much of the effect of tel1 deletion seen in this study is attributable to relieving the effects of nonphysiological Tel1 hyperactivation.

As suggested, we have further included this point at the end of this section (Lines 551-564).

5. The log-fold difference plots do not provide any visual correction for the changes in absolute DSB levels. For example, since total DSB frequencies have gone up in tel1 mutants, then the log-fold difference of the tel1 map relative to the wild-type map may give a misleading impression: regions that have experienced an increased DSB frequency but to a lesser degree than the average increase will look like their DSB frequencies have gone down instead. One problem is that the CC-seq maps are not calibrated to yield absolute DSB levels; they are only normalized relative to the sample mean (hits per million mapped reads). The caveats of using non-calibrated maps should be explained, and if possible, some indication of how to visually correct the log-fold difference maps should be provided. For example, a line or shaded area could be added to the plots to indicate an estimate for what log-fold value corresponds to no change in absolute DSB frequency; this could be estimated from the Southern blotting data (not optimal, but sufficient for the purpose here). This issue is particularly relevant to the discussion on lines 464-469, which emphasize the magnitude of the effect of Tel1: it seems quite possible that this magnitude is mostly or completely an artifact of using a sae2 background for these experiments.

Based on Southern blotting, our best estimate at this stage of our studies is that either *ndt80Δ* or *tel1Δ* separately increase DSB formation about 1.5-2 fold—although this is likely to be at relatively strong hotspots which may not be representative of the global change. We have also explored using a spike-in control to be able to estimate fold changes (as discussed with reviewer 1), but are not confident in being able to apply a correction due to relatively high variance. For these reasons, we prefer to focus on relative (rather than absolute) changes in the distribution of Spo11 activity because these changes are robust and unambiguous.

Nevertheless, the reviewer raises an important point relating to data interpretation and we have discussed the issue and the caveats raised in the main text when we first introduce these datasets (Line 319-328). We don't, however, think this point is relevant to the section highlighted in the discussion by the reviewer because in that section we are specifically discussing differences in the magnitude of the effect on the distribution that arises ±Ndt80 vs ±Tel1.

6. Throughout: for bar graphs, it is becoming standard practice to superimpose individual measurements when the number of these is small, so it would be good to do this. Likewise for the interference plots (Fig. 1m and similar). Also, SEM is shown throughout, but this is rarely an appropriate choice to display experimental variation because it can be visually misleading, especially when the sample size differs between test conditions as it does here. SD should be used instead.

All plots have been updated to include all individual data points as hollow circles superimposed on the bar and with standard deviation whiskers replacing SEM.

Minor points

1. Line 89: Mek1 is more accurately described as a paralog of Rad53, not an ortholog

We have changed ortholog to paralogue (Line 92)

2. Lines 119-124 and Figure S1a: In the introduction it is stated that Tel1 activity promotes checkpoint activation and delays Prophase exit. Figure S1a shows the same model, however the experimental data shown in Figure 1b and S1b-d only shows the rescue of Prophase exit timing in a sae2Δ background.

There should be a rationalization of why repair mutants like Rad50S or sae2Δ mutants need to be used to measure the change in Prophase kinetics.

We are using *sae2Δ*, not so that we can measure changes in prophase kinetics, but, rather, so that we can measure changes in DSB accumulation. In this section we specifically introduce the fact that Tel1 alters prophase kinetics in the *rad50S* background (Usui paper), and thus might be having a similar impact in *sae2Δ* cells, which we then go on to test. From these analyses it seems clear that the accumulation of unresected DSBs triggers a Tel1-dependent checkpoint arrest, and thus potentially reduces the window of opportunity for DSB formation in the *sae2Δ tel1Δ* strain relative to the *sae2Δ* control.

At the end of the second paragraph of the discussion (Line 423-425) we have added the sentence: “Importantly, due to the unresected DSBs that accumulate, Tel1’s role in meiotic checkpoint control is much greater in sae2∆ cells than it is in wild-type cells, thereby significantly altering prophase kinetics.”

3. Line 122: The way the text is worded makes it sound like this is the first demonstration of the division delay in the sae2∆ mutant, but this has been known since the original characterization of SAE2 (McKee and Kleckner 1997). Suggest rewording and citing the earlier work.

We have amended the text and cited this earlier work (Line 126-127).

4. In Figures 1, 4 and S7 it could facilitate interpretation for the reader if the color for wild type was different. Overall, the consistent color scheme is very helpful, but it is hard to distinguish the different blue tones.

WT and *tel1∆* colour was changed to a different tone keeping the colour scale consistent with blues for *TEL1+* and yellow/brown for *tel1∆*. We hope these colours are now different enough to differentiate each strain.

5. Line 150: not clear what the word “both” refers to (both hotspots in the region? but there are three hotspots quantified…). Please clarify.

This was a grammatical error. We have removed “both”. (Line 159)

6. Line 187: I don’t understand the rationale for averaging the 6 and 8 hr time points. As the text says, DSBs and DCs continued to accumulate from 6 to 8 hrs; since these time points are demonstrably different from one another, there does not appear to be any justification for averaging them.

We have amended the text (Line 195-200) to avoid confusion here. Whilst it is true that the DSB and DC values increase over the timecourse, the difference is often quite little, and our experience is that taking an average of the 6 and 8 hour timepoints helps to reduce technical error in the measurements being made. We have additionally cited our prior work that used the same method. In addition, as suggested by Reviewer #1, we have included a supplementary panel (Fig S1m) that measures DSB interference over time (4,6,8 hours) and referred to this analysis in the text. (Line 217-219).

These single-timepoint data have more variance but show the same pattern as is obtained when averaging 6 and 8 hours together, which we feel justifies our decision to average those timepoints. Only 4 hours looks possibly different (at least in the *sae2∆ ndt80∆* sample), but this timepoint has the highest variation in the interference measurements, so we would question drawing any strong conclusions.

7. Lines 235-237 (“over short distances Spo11 DSBs failed to display interference”) and Lines 265-267 (“Tel1-dependent DSB interference acts over both short and medium scales”) The formulation could be improved here: if I understand correctly there is interference (mechanistically) mediated through Tel1 over short and long distance, however on short distances this fails to be measured as interference because of the underlying clustering of DSBs.

Indeed, this is exactly our model. We have rewritten this closing paragraph (Line 247-250 and 283-287), incorporating some of the reviewer’s suggested language to hopefully make this conclusion easier to follow.

8. In Figure 3 g-h the authors show the local enrichment of DSBs when Ndt80 is present in the absence of Tel1. The overall correlation is convincing but there are notable exception in chromosome 4 (and maybe also a bit in 14). Is there any particular explanation (genomic context?) why the correlation might be poorer in these regions?

Individual correlations for each chromosome are quite variable and often quite low—for example chromosomes 1,3,4,7,11,12,13 are all 0.4–0.5. As such, we infer that Rec114 association may only be one component of the correlation. As stated in the text, the correlation with centromere proximity is also likely to be a major contributor. We have updated the section heading here to help emphasise this point: “**Prophase arrest redistributes DSBs away from centromeric regions and regions of early Rec114 association**” Line 289.

9. Figure 4g the legend for the log2 fold change states -1.5 on both ends.

This figure was amended.

10. Line 317. “We further propose that it is this effect that drives the negative DSB interference (DSB clustering) that we have measured over short distances” this sentence is a bit unclear to me and requires some elaboration.

We have substantially modified this paragraph, hopefully making this latter point clearer: “Given that NDT80 status also alters measurements of DSB interference over short distances (above) we propose that it may be transient enrichment of Rec114 within early regions that drives the negative DSB interference (DSB clustering) that we have measured when Ndt80 is present (above).” Line 351-358.

11. Line 349-352. This conclusion is difficult to interpret without the underlying manuscript.

The manuscript is still in preparation, but will be placed on bioRxiv once ready. The core observations (that a process of localised DSB inhibition forming around each DSB can generate complex genome-wide patterns of fold change) have been presented at recent

Gordon, EMBO and UK meiosis meetings over the past few years. We have expanded our description here to provide additional information: Line 390-395. “Ongoing work in our laboratory supports the view that that at least some of these Tel1-dependent and chromosome-specific patterns of fold change can manifest via distance-dependent DSB interference arising around each DSB as it forms—collectively generating spatially patterned changes in the population-average frequency of DSB formation within hotspots (LLR and MJN; manuscript in preparation).”

12. General: throughout the manuscript the heterogeneity of the assayed cultures and the limitations posed by population-based assays are an important aspect of the model. I therefore think the authors should discuss in more detail how the assayed timepoints compare between the different genotypes.

We have updated the model figure to hopefully clarify our meaning of heterogeneity of active domains across the cell population and how this can impact measurements of DSB interference. We always compare the same time points to one another across the genotypes (an average of t=6 and t=8 hours in order to reduce technical variation). We do not feel confident to draw conclusions that might be made from differences through time due to the greater technical variation in measurements of interference at single timepoints.

13. Given the substantial quantitative variability between loci for the effects of the tel1 and ndt80 mutations when analyzed by Southern blotting, it was a bit surprising that the whole-genome data was not analyzed at these specific locations (at least, those locations present in the strains used for CC-seq).

The CC-seq data only provides estimates of relative DSB frequency at each hotspot, and does not measure DC formation (unlike gels), thus it is unclear to us what type of CC-seq analysis would be of merit at these specific loci.

14. Line 677: Thank you for providing the scripts. Please add a mention here that tables listing the called hotspots are also provided on the Github deposition. (If this is mentioned elsewhere, I missed it.)

We have added the GitHub address of the Hotspot folder to the extended methods section. Line 924-926.

REVIEWER 3

Reviewer #3: In their manuscript “Meiotic prophase length modulates Tel1-dependent DNA DSB interference”, López Ruiz and colleagues analyze effects of checkpoint kinase Tel1 and prophase I exit factor Ndt80 on local meiotic DSB levels and interdependence as well as DSB global placement. Consistent with earlier findings, they report that Tel1 mediates delayed meiotic progression in a mutant that blocks DSB resection (sae2D), but also prevents the

clustered formation of DSBs along the same chromatid in the same cell. Whether Tel1's role in minimizing closely spaced DSBs is related to its role in extending the time spent at the DSB stage or whether Tel1 performs this function independent of timing issues has been unclear. Here, the authors use the ndt80D mutation to arrest sae2 and sae2Dtel1D cells indefinitely in prophase I. They find that extended prophase partially suppresses the formation of closely spaced DSBs in absence of Tel1 while at the same time ndt80D enhances DSB formation synergistically with tel1D. The authors show that genome-wide, Tel1 is especially important for limiting DSBs in regions that load Spo11 cofactor Rec114. Enhanced DSB formation in regions occupied early by Rec114 is diminished when the accelerated prophase exit in tel1D sae2D is blocked by ndt80D, suggesting that Tel1 limits DSBs only in part via its role in delaying meiotic progression, and that non-checkpoint functions also play a role.

Overall, these findings are quite intriguing, and the data are of excellent quality. My main concerns are with the presentation of the findings.

First, there is a strange disconnect between local and global effects. The effects on DSB enhancement and DSB interference are determined by distance, but only limited attempts are made to connect these to the global effects. Do the global effects on DSB levels and placement shown in Figures 3 and 4 predict local effects at DSB sites?

We favour the converse view—that local effects of DSB interference can create unanticipated chromosome-scale patterns of fold change. Detailed exploration of these effects is the subject of a manuscript currently in preparation.

Could the authors point out where the DSB sites explored in Figure 1,2 , S2,3,4 are located, if possible in a main Figure?

We have added a diagram to each figure highlighting the position of the loci being studied.

Is there a connection between hotspot-specific effects observed in Figures 1, 2 and S2-4?

Overall, the effects are broadly similar at all the hotspots studied: Increases upon either *NDT80* or *TEL1* deletion; low (or negative) interference in the absence of Tel1; increased measured interference upon deletion of *NDT80*. Any locus-specific differences may be caused by the local genomic context and or intrinsic amplitude of DSB formation at or around that site.

Can differing effects be explained by their localization along the chromosome?

Potentially, yes. However, overall, the effects at each hotspot are quite consistent (as summarised above), the only major exception in our view being the retention of negative interference between hotspots NO (panel S4m), which we speculate may in some way be related to the fact that this hotspot is only detectable in the absence of Tel1.

Second, while struggling with Figure 3, I found myself jumping ahead to Figure 4 to find out about the effects of tel1D and tel1D ndt80D. I understand the rationale of keeping the same order of presentation from the earlier figures (sae2 vs sae2ndt80 followed by sae2 tel1 vs sae2 tel1 ndt80), but the reader is told about ndt80D effects without knowing about tel1D effects. The Abstract (L26-28) actually summarizes the findings in this order.

I assume that the authors want to front load the most intriguing findings in Fig. 3g,h, but maybe there is a compromise? Would it make sense to combine into a single Figure data in Fig. 4 d,e (comparison of sae2D/tel1Dsae2D) then add the ndt80D effects (Fig. 3 d,e)?

We thank the reviewer for their suggestion. We agree it is tricky because there are multiple comparisons that one may wish to make. On balance, we prefer to retain the current organisation because it helps separate the two different effects: Figure 3 concerns the ±Ndt80 effect, which is stronger in the absence of Tel1 presumably due to Tel1's role in checkpoint activation altering prophase kinetics in the presence of Ndt80. By contrast, figure 4 gives a glimpse of the ±Tel1 effect on genome-wide scale which we infer is at least in part due to its role in DSB interference—and demonstrate that despite the strong effects that ±Ndt80 can have (in Fig 3), the ±Tel1 effect is in fact largely unchanged ±Ndt80. We are in the process of preparing a manuscript that describes these Tel1 effects in more detail—but this is beyond the scope of this study to be included here.

Also, can you show all effects along the same chromosome? It is frustrating that Figures 3 and 4 focus on different chromosomes (VII and IV, respectively) so it is left to the reader to put together a complete comparison of genome-wide DSB effects.

As suggested, we now show Chromosome 4 in both figures.

Overall, the Introduction and Results are well written, but the Discussion feels like a collection of ideas instead of putting all findings into context. Also, the discussion fails to consider alternative explanations:

-Could the reversibility of DSBs that carry Spo11 at their ends play a role, i.e. that in ndt80D some double cuts get resealed?

We have no evidence to support the view that DSBs that accumulate in sae2D cells are capable of undergoing religation—at least not at a measurable frequency, thus we see no need to discuss this possibility. Even if this were the case it is hard to imagine why this would specifically affect inter-hotspot double-cuts (which are themselves simply a pair of adjacent coincident DSBs) and not all DSBs.

-Or could additional DSBs formed in ndt80D tel1D not exhibit negative interference because DSB clustering is suppressed by a factor that acts redundantly with Tel1 at later stages (NB Mec1 affects resection of later DSBs in this way, Joshi et al, Mol Cell, 2015)?

We thank the reviewer for the comment and have included this idea in the discussion. Line 586-591.

Finally, a major conclusion from this work seems to be that DSB distribution and frequencies observed in sae2D/rad50S represent an underestimate (previously suggested by Borde and Lichten, Science, 2000) and that actual DSB frequencies should be derived from sae2D ndt80D?

I'm not sure there is any "perfect" way or system to assess DSB frequencies globally. Each methodology (Southern blotting, Spo11-oligos, S1-seq, END-seq, ChIP-seq, ssDNA-ChIP, CC-seq etc) and choice of genotype to assess it in (be it wild type or mutant) has its own pros and cons. Whilst *NDT80* deletion both increases DSB formation and alters the genome-wide distribution, our observations suggest that these effects are more pronounced in mutants such as *tel1*∆ *sae2*∆ that exit prophase more swiftly. For example, there is only a limited effect on the distribution in sae2D vs sae2D ndt80D cells (Fig 3f.). Moreover, one could easily argue that arresting cells for prolonged periods via Ndt80 deletion provides more time (opportunity) than might arise during a normal wild-type meiosis.

We have added a section in the discussion (Line 518-521) that we hope covers the reviewer's point and cites the original sae2D/rad50S observation: "It has been suggested that DSB distributions and frequencies arising in sae2∆ or rad50S cells represent an underestimate of the wild-type patterns{Borde et al., 2000, #19045}. However, given that deletion of NDT80 had only a relatively minor impact in the presence of Tel1 (Fig 3f) suggests that any underestimate is likely not due to premature exit from meiotic prophase."

Is there any support for that conclusion from comparison with crossover/non-crossover or WT DSB maps?

As mentioned above, the global effect on DSB patterns upon *NDT80* deletion is subtle in the presence of Tel1, so we see no reason to think that this will change any relationships that have already been characterised between DSBs and recombination.

If you think that that is the case, you should say so and provide a revised estimate of the DSB map and frequencies. If not, then you should clearly state whether or not the DSB potential is fully exploited in WT cells.

I don't believe that we emphasise the effect raised by the reviewer as a major conclusion from our work. Rather, our major conclusion is that *NDT80* deletion appears to suppress subpopulation effects on short-range coincident DSB formation—effects that appear particularly strong in the absence of Tel1-dependent DSB interference. Our study does not measure whether or not DSB potential is fully exploited in wild-type cells—rather, our study is primarily concerned with characterising effects on DSB interference.

As a more general point, the authors frequently state mutant phenotypes without explaining how these phenotypes are relevant to the wild-type situation, and the manuscript would be stronger if they could clarify the relevance to the WT.

We have added a section in the discussion about how the effects we observe in the *sae2*D background may compare and inform effects that arise in wild-type cells: (Line 554-564) "For example, if like in mitotically dividing cells…should be interpreted with such differences in mind."

Here are some specific points. Throughout the manuscript: It is not clear why the authors use the term Spo11-DSB? It's fine if it is supposed to indicate that DSBs in sae2D still carry Spo11 at their 5' end. But does the reader really need a constant reminder that this is a meiosis paper and DSBs are made by Spo11?

We have read through the manuscript and use both "Spo11 DSB" and "DSB", the former sometimes for a reminder and/or added emphasis or context. We have removed a few additional "Spo11" that might be considered superfluous, but we see no reason to remove all instances of this term—after all, we are characterising Spo11-DSB formation, not any other form of DSB that may arise. [Note: out of 255 instances of "DSB" there are now 31 instances of Spo11-DSB or Spo11 DSB, which we do not think is excessive].

L36: "Pairing…facilitates homologue alignment" – could you add "along the length of homologues", without that it's really obscure how pairing and alignment are different.

We have revised this sentence to make it simpler. (Line 39-40).

L63: What are "proactive features"

We previously introduced this term to contrast with "reactive" modes of DSB regulation that happen downstream (Cooper et al 2016).

L121: Why not refer to the time of 50% of max divisions so the reader knows where to look for the indicated 3 hour delay. Maybe also point out that sae2D is a mixed delay/arrest whereas the fraction of arrested cells is much smaller in absence of Tel1.

We feel that the 2-3 hour delay is readily apparent from the curves presented in Fig 1b, and the precise extent of the delay is not relevant to the point being made. We have additionally referred to the delayed second nuclear division in *sae2*∆ (and the rescue upon *TEL1* deletion). (Line 127-128)

L133: Please define in the introduction what you mean by "DSB (forming/formation) potential" and use one term consistently throughout the paper. The idea that (during wild-type meiosis ?) some DSBs have potential but do not fulfill that potential is really not that obvious. The term currently appears in the Abstract and then again in the Results section.

We have clarified this point: (Line 140-143) "For our purposes, we define "DSB-forming potential" as the pre-activation (maturation) of subchromosomal regions within a cell such that

they gain the potential to catalyse DSB formation. We have hypothesised that maturation involves some kind of upstream proactive, priming process (Garcia et al 2015; Cooper et al 2016).”

L150: Not sure what “both” refers to

This was a grammatical error. We have removed “both”. (Line 159)

L195: “even though Tel1… DCs inhibited” is redundant

Although this could be removed, we purposely added it to help reinforce to the reader that positive interference is not detected despite DCs being inhibited. We prefer to retain the original text for this reason.

L241: Could that be: “Tel1 controls DSB interference over medium distances independent of Ndt80”?

We have modified in a similar way to as suggested: “Tel1-dependent DSB interference over medium distances is unaffected by *NDT80* deletion” (Line 252-253).

L269: Again, could you rephrase that subheading to indicate how the mutant phenotype informs us about the WT, rather stating the mutant phenotype?

Based also on other feedback, we have modified to: “Prophase arrest redistributes DSBs away from centromeric regions and regions of early Rec114 association.” (Line 289-290)

L370: Sae2 is referred to as an Mre11 activator, but ref 29 (Keeney, Kleckner) seems to be incorrect, and the other two papers (McKee; Prinz) do not speak to the mechanism by which Sae2 affects resection. Ref 25 (Cannavo and Cejka. 2014) might be more appropriate if the authors want to refer to Sae2’s activity.

As suggested, we have amended these references to instead include studies referring to Sae2’s role in activating Mre11 at DSB ends and in meiotic cells (Cannavo et al 2014; Cannavo et al 2018). Line 423.

L372: If tel1D and ndt80D increased Spo11 activity independently, as stated, then both single mutants should result in increased DSBs. But that is not the case, for sure not in Fig. 1f-h. It looks more like a synergistic effect, where ndt80D affects DSB levels only in a tel1D background (Fig. S3f is the exception where ndt80D does increase the levels of DSBs by itself). The conclusion should be that Tel1 becomes even more critical in limiting DSBs in an ndt80D background.

We regret the oversimplified description in the text which was an attempt to summarise the main effects observed across the many loci we have measured. Indeed, in most cases, the largest effect of deleting *NDT80* is in the *tel1*Δ background. We have amended the text to hopefully make the point emphasised by the reviewer clearer: “When considering total DSB levels, whereas *TEL1* deletion alone increases DSB formation, the effect of deleting *NDT80*—and thus of extending prophase—was generally stronger only in the absence of Tel1 (**Fig 1f-h; Fig S2d-f; Fig S3d-f**), suggesting that Tel1 activity becomes even more critical in limiting DSBs in an *ndt80*Δ background.” (Line 427-30)

L390: The sentence about detection of positive interference comes out of nowhere and needs to be embedded in the argument.

These two sentences lead directly from the section about double cutting and are setting up the background to the next section about interference not being detected in the presence of Tel1 over short range. We have added “positive” as stated by the reviewer to perhaps make this sentence easier to understand: “Intriguingly, in our prior work, although we clearly demonstrated Tel1-dependent DSB inhibition, over very short inter-hotspot distances **positive** interference was not detected in the presence of Tel1” (Line 447-451)

L395: “these effects”: It’s unclear what effects you mean and how this is related to the previous paragraphs

We have clarified this point in parenthesis. (Line 453-455) “These” referred to the observations described within the prior two sentences: “We previously proposed that these effects (the inability to detect positive interference—despite clear evidence that Tel1 impedes double cutting—and the observation of negative interference in Tel1’s absence) can arise…”

L400: “If the activation step that limits total DSB potential…” I don’t understand this sentence. Isn’t it evident from the fact that different cells form DSBs in different places that “active domains vary in their chromosomal location across the cell population”?

In our thinking, activation refers to the priming of a domain that, potentially, encompasses dozens of DSB hotspots, where only some (or none) of which will form. In this model, activation is a prerequisite for DSB formation, but it does not guarantee a DSB will form. DSB formation itself will be affected by other features of the individual hotspots, their local chromatin environment, and perhaps other features—including reactive negative regulation by Tel1. We have substantially revised this section to hopefully help clarify our point: (Line 460-471).

“We propose that pre-activation of a region—upstream of DSB formation—is one such limiting step, creating—at any given chromosomal region—a heterogeneous mixture of domains that can or cannot initiate DSB formation across the cell population (Fig 5a, top). Critically, because in this model DSB formation is restricted to happen only within those domains that have undergone pre-activation, such heterogeneity will give rise to underestimates of DSB interference (Fig 5a, bottom).

In wild-type cells, when Tel1 is active, the formation of such pre-activated subdomains is likely to help disperse a limited amount of DSB potential across the genome. However, in tel1Δ, the absence of localised negative regulation will permit efficient coincident cutting by Spo11 at all DSB hotspots located within any local pre-activated region—therein detected as negative interference{Garcia et al., 2015, #16838} (Fig 5a, bottom)."

The real question seems to be whether all cells have the same distribution of DSB potential along their genomes, and only the activation is different in each cell, or the DSB potential already is different in different cells. The discussion should explain how findings in this paper contribute to answering this question, specifically: what are the contributions from sufficient time spent in prophase (mediated by Ndt80) versus other regulatory functions (mediated by Tel1).

Based on our findings (changes to the way interference calculations play out ±NDT80), we have proposed that increased time increases the potential for regions to mature/prime/activate—whatever word one prefers to use. We feel we have described this idea clearly within the manuscript including in the abstract and here in the discussion.

L414: Please explain why you think that eliminating NDT80 makes the population more homogeneous?

Because of the increased time cells can spend in prophase. To further clarify our point, we have added: "Thus, in this model, the extension of meiotic prophase caused by *NDT80* deletion homogenises the population because it gives more time for chromosomal subdomains to mature and become active, reducing subpopulation effects." (Line 478-480)

The sae2 tel1 population seems to be as homogeneous as the WT – wouldn't non-homogeneous mean that a subpopulation of cells arrests or the population is biphasic?

That would be one possibility. Another (that we favour) is that it is the pre-activation of chromosomal domains—priming them for the potential to form a DSB—that is nonhomogenous. This is the same idea we proposed in Garcia 2015.

If you mean that all potential DSB sites get an opportunity to become activated due to the extended duration of prophase I, then that should be stated clearly.

We hope that the revised text in this section (copied above) clarifies this point.

L419: I thought that this is one of the questions that this paper tries to answer: whether Tel1 plays a dual role in DSB interference and checkpoint activation? I think that point would deserve some elaboration.

It is already known that Tel1 plays a dual role. This was not what we were trying to answer.

Rather we are studying the interplay between the two roles and how this affects interference measurements. We see no need to elaborate this point.

L466: Please clarify this section. I'm assuming this should say: tel1D results in increased DSB formation in certain chromosome regions independent of the presence or absence of Ndt80?

To clarify, we have added: "...on the DSB distribution…" in the following revision: "...deletion of *TEL1* elicits a much stronger effect on the DSB distribution, which is detectable both in the presence and absence of Ndt80 (**Fig 4f-g**)." (Line 532).

Figure 1b: Please show here or in the Supplement that you have confirmed that all mutants arrest in the ndt80D background.

As expected, none of the *ndt80Δ* strains we have analysed undergo meiotic divisions even after 24 hours. We have included this information in Fig 1b.

Figures 3f-g and 4f-g: Can you clearly mark the chromosome ends? Or make the background black? The surrounding color is very similar to the shading of 0-fold changes.

We have updated these figures as suggested.

Figure 5: In 5b, ndt80D should be written in lower case letters.

We have corrected this typo.

I feel the loops in cells 1-4 in the upper half of the figure could be easily be omitted or only shown once, providing more space for the all important lower half of the figure.

We have amended the final figure to simplify following comments from the other reviewers.

Legend Figure 5: It should say upfront in the Figure that this model refers to short-distance interference.

We added: "at short range".

The phrase "lower-than-expected calculations of DSB interference" is a real brain twister: (Positive) interference refers to instances where the observed outcome is lower than the (calculated) expected.

Aren't you simply referring to underestimates?

Indeed, as suggested, we have replaced this phrase with "underestimates".

Also, how can the DSB frequency be underestimated when DSBs are a measured entity?

We were attempting to refer to the frequency of DSB formation within the preactivated fraction of loops (itself an unknown quantity). i.e. If all domains are not activated (let's say it is only 50% for the test region) then, for a given population-average DSB frequency that is observed (let's say 10%) the DSB frequency per activated loop will be greater (20%).

Based on feedback from other referees we have revised figure 5 to hopefully make these points clearer.

It seems the calculations only work out if there are 2 clustered DSBs in Cell 3, but it is unclear why Cell 3 in the tel1D ndt80D mutant receives two cuts while Cell 1 receives 3?

Figure 5 has been substantially revised to simplify the comparison we are making.

Have all data underlying the figures and results presented in the manuscript been provided?
Large-scale datasets should be made available via a public repository as described in the PLOS Genetics data availability policy, and numerical data that underlies graphs or summary statistics should be provided in spreadsheet form as supporting information.

Reviewer #1: Yes

Reviewer #2: Yes

Reviewer #3: Yes

PLOS authors have the option to publish the peer review history of their article (what does this mean?). If published, this will include your full peer review and any attached files.

If you choose "no", your identity will remain anonymous but your review may still be made public.

Do you want your identity to be public for this peer review? For information about this choice, including consent withdrawal, please see our Privacy Policy.

Reviewer #1: No
Reviewer #2: No
Reviewer #3: No
