# Supplementary material for: Meiotic prophase length modulates Tel1-dependent DNA double-strand break interference
Source: PLoS Genet. 2024 Mar 1;20(3):e1011140. doi: 10.1371/journal.pgen.1011140 (PMC10936813; doi:10.1371/journal.pgen.1011140)
Supplement: Attachment — Submitted filename: Second reviews_Response.docx [file pgen.1011140.s014.docx]

2023-12-13: Response to reviewer and editorial comments.

---------------------------------------------------------------------------------------------------------------------------

Editor’s comments:
Thank you very much for submitting your Research Article entitled 'Meiotic prophase length modulates Tel1-dependent DNA double-strand break interference' to PLOS Genetics.

The manuscript was fully evaluated at the editorial level and by independent peer reviewers. While two of the reviewers were satisfied with the revision, Reviewer #2 was not, and I regret to say that I agree completely with their concerns. Based on this, we will not be able to accept this version of the manuscript, but we would be willing to review a much-revised version. We cannot, of course, promise publication at that time.

Reviewer 2 points out that several of the comparisons made in the manuscript, in particular those centered around Figure 2, are not statistically significant. This being the case, no further conclusions about relationships can be made. That's is, bottom line. As stated by the reviewer, in such cases all that can be said is that one set of values is not significantly different from the other; this negates further conclusions based on differences but also further conclusions based on similarity. I strongly suggest that the manuscript be revised as indicated by this reviewer, including the "Additional points" that refer to two other concerns regarding the manuscript.

In addition, now that the individual data points are available, it is clear that there are substantial technical problems, in particular with DSB frequencies in *sae2∆ ndt80∆ tel1∆* strains from *FRM2*-probed blots, where only two replicates are present and the difference in values between the two is greater than three-fold. This issue was pointed out in previous reviews, and there certainly has been sufficient time between submissions to address it. While I am not going to insist that additional replicates be done (perhaps technical replicates by reprobing filters with the "other" probe?), you must know that the data in their current form really do compromise credibility. Is there any way that you can begin to address the source of this variability? For example, in Figure 1, do frequencies of DSB II from MXR2-probed gels agree with those on HIS4-probed gels? In Figure 2, do frequencies of DSBs at leu2::hisG on CHA1-probed gels agree with those on FRM2-probed gels?

A: We thank you for the very helpful comments, which led us to reappraise the data that we were aggregating in our analysis of interference in Fig 2. In our revision we have added two additional repeats, but also removed data from one blot that we identified as being a clear outlier in terms of estimated DC and *leu2*::*hisG* DSB frequency (explained below).

Importantly, exclusion of data from this one blot is far from arbitrary, but rather justified by what appears to be an unusual pattern of signals on the blot that are not expected. Specifically, we identified a doublet of the parental full-length chromosomal band plus additional DSB signals in the lane in regions not predicted. Our working explanation is that the probing of this blot inadvertently cross-reacted with a second chromosome of similar size to chromosome III leading to these additional bands, and importantly, thus to a reduction in the relative proportion of lane signal in the measured Spo11-DC and *leu2*::*hisG* DSB bands. Notably, the original analysis of these data reported values that were about half those on other blots, consistent with this interpretation. However, rather than retain the data (and perhaps multiply the values by two in an attempt to correct this probing effect), we have excluded this blot and replaced it with two new *sae2*∆ *ndt80*∆ and *sae2*∆ *ndt80*∆ *tel1*∆ datasets, which also means the total number of biological repeats reported for all strains in this figure is now three.

Please refer to the attached annotated figures (below) of the revised collated data, plus the blot (TC12, PFGE30) that we have removed from the resubmission. We apologise for this prior oversight, which was a major source of the high degree of variation observed in Fig 2c,d,f,g. All *P* values and comparisons have been updated accordingly.

At any rate, as a minor point, current convention is that SD should not be used for error bars when only two values are present--in such circumstances, error bars should report range.

A: All main figures now have n>=3. In supplementary figures, when n=2 for some loci, given that individual data points are shown as open grey circles, the range of data (which is less than the SD when n=2) is clearly visible, thus a range bar wouldn't add any additional information. For these reasons, to maintain consistency, we prefer to use error bars to indicate SD in every panel (and this is clearly stated in each legend).

Finally, unless I missed a file, the data underlying each graph has not yet been reported in an accessible way.

Data underlying each figure panel should be reported in a separate table that can be readily identified as belonging to that figure panel. If a point on a graph represents the mean of two or more values, then the table should contain those individual values as well as the mean. The current Excel file is impenetrable, and much of the data reported in the paper are missing from this file (c.f. panel 1b, 1f, 1g, etc.).

A: A collated data file that underpins the information plotted in each graph has been added to the revised submission: NDT80_Submission_Data_01.xlsx

In addition, we have made a very small number of typographic changes to correct formatting and text errors, and to improve clarity (highlighted throughout the text file).

------------------------------------------------------------------------------------------------------------------------

Reviewer #1: The authors responded satisfactorily to all my comments. I recommend accepting the revised manuscript. I appreciate the authors' efforts to conduct technically challenging experiments and draw robust conclusions successfully. Here are my final minor comments.

We thank the reviewer for their positive comments.

I wonder if plotting all DSB interference measurements as a function of the distance between hotspots could further emphasize the robustness of the authors' conclusions.

A: We thank the reviewer for this suggestion. We have added this collated analysis to Fig S4e. Whilst the number of data points is quite low, in both NDT80+ strains there is a positive correlation between interference and inter-hotspot distance that is substantially weakened/lost in the *ndt80*∆ strains. However, with such few data points, we are cautious as to whether these fit a linear relationship over distance (as measured by Pearson). It may just be that the close-range hotspots (below 15 kb) act as one group (and the longer-range hotspots as another.

How about activating one more domain on the left in Figure 5a to match the text below "Probability of domain activation = 0.5"?

A: Many thanks for spotting this. This was an oversight when redrawing the figure, which was indeed meant to show 50% of loops in the active state. We have amended the figure.

---------------------------------------------------------------------------------------------------------------------------

Reviewer #2: The authors have addressed most of the concerns raised in the previous reviews. However, there remain concerns about conclusions drawn where differences are not statistically significant. As noted in prior review comments, the study is underpowered to make some of the conclusions the authors wish to draw. The authors have argued out of providing the additional replicates needed to resolve these issues, which is fine, but they must accept that they can’t make claims that do not have statistical support. Note that switching post hoc to using one-sided p values (as mentioned in the response to reviews) would not be appropriate, and cannot be used to justify making claims that lack statistical support.

Examples:

Lines 259-261: the difference between sae2 ndt80 tel1 and sae2 tel1 is not statistically significant. Therefore, no conclusion can be drawn here, and the data most definitely do not suggest an additive relationship, as asserted. The observation can be described, but the claim that the data suggest the additive relationship needs to be deleted. The fact that DSBs at another hotspot (leu2::hisG) do not show any similar trend further undermines confidence in the point the authors wish to make.

A: Whilst we consider it likely that the relationship is additive, as part of the revision to this section (to take into account the revised numbers), we have deleted the comment about the additive relationship. (Lines 257-267.)

Line 267: again, not statistically significant (p = 0.12). Looking at the data here (Fig 1f), two replicates showed an increase and one replicate showed a decrease relative to the mean for sae2 tel1. There is simply not enough information here to draw a conclusion. Throwing in “albeit variable” to qualify the claim of an increase is not appropriate. The claim needs to be deleted.

A: We have revised this section taking into account the suggestions and also based on the revised quantification/repeats that are now included. Due to the removal of the erroneous dataset, and addition of another repeat, this increase in the *sae2*∆ *ndt80*∆ *tel1*∆ mutant is now statistically significant (P=0.00009). (Lines 269-278.)

Line 268: this doubles down on the claims above about there being increases in both the single cut and double cut frequencies. The statement here is simply not supported by the data. There really isn’t any need to make this claim anyway, so it can be safely deleted.

A: We have revised this section taking into account the reviewer’s suggestions and based on the revised quantification/repeats that are now included. (Lines 269-278.)

Lines 275-281: The data provide no evidence to support the conclusion that DC frequencies are “modestly increased” by ndt80 deletion in the presence or absence of TEL1, as claimed. This needs to be deleted. The conclusions about interference are mostly fine, except that the the “weakening” when comparing sae2 and sae2 ndt80 is again not significant.

A: We have revised this section taking into account the reviewer’s suggestions. (Lines 280-284.)

Additional points:
Lines 249 and 285: I find it confusing to couch the conclusions here in terms of “underestimating” interference. If I am following the argument, the point is that the numerical value for the interference calculation is lower (more negative) in the presence of NDT80 for short distances, and gets larger (closer to zero) in the absence of NDT80. If so, then say that. The problem with saying that the interference is “underestimated” is that a more negative value actually means STRONGER (negative) interference than a value close to zero. Interference can be either positive or negative, and the further you are from zero, the stronger each type is. It is not really until Fig 5 (Discussion) that this idea of underestimation of interference is properly explained. I’d suggest using different wording in the earlier sections of Results.

A: We have revised this section to hopefully make our summary less cryptic. (Lines 247-253 and 286-291.)

Lines 390-395 and 533-539: It’s not appropriate to make assertions like this based on unpublished data. Whether this information has been presented at conferences (as stated in the response to reviews) is immaterial. These sections need to be deleted and this paper constrained to discuss what is actually presented here: the authors can make these other points in the other paper when it comes out.

A: We have made deletions to these sections. The manuscript describing these analyses remains in preparation at this time. (Deleted text entirely at 394-396 and partially at line 536.)

We retained the single sentence in the discussion (Line 533-536): "We hypothesise that at least one component underpinning these strong Tel1-dependent changes is the genome-wide effect of DSB interference (LLR & MJN, manuscript in preparation), which is robust to changes in the length of meiotic prophase."

Line 611: GEO accession is still described as pending

The GEO accession is now live and publicly accessible: https://www.ncbi.nlm.nih.gov/geo/query/acc.cgi?acc=GSE245327
We have added this information to the manuscript.

Reviewer #3: The authors have made revisions that appropriately address reviewer comments.

We thank the reviewer for their helpful comments during the review process.
